# Supplementary material for: Barriers and facilitators of successful deprescribing as described by older patients living with frailty, their informal carers and clinicians: a qualitative interview study
Source: BMJ Open. 2022 Mar 28;12(3):e054279. doi: 10.1136/bmjopen-2021-054279 (PMC8961139; doi:10.1136/bmjopen-2021-054279)
Supplement: Supplementary data [file bmjopen-2021-054279supp001.pdf]

Qualitative interview: Clinicians: v.1.3

Qualitative interview: Clinicians	
**Topic**	**Questions**
**Deprescribing: Process**	Can you please talk about the last time you stopped a patients' medicine (or recommended it)? Probes: When did it happen, why, how and which medicine(s)? Who initiated the discussion?
	What information and support did you provide this patient to make him/her feel comfortable about stopping these medicines? What went well and what could have gone better?
	What thoughts and/or concerns did the patient discuss during the discussion to stop this/these medicine(s)? What options were there for the patient and how was the decision made?
	Did this patient experience any issues as a result of stopping this (these) medicine(s)? What strategies did you use to help him/her overcome these difficulties?
	Do patients ever ask you to have one or more medicines stopped? Can you tell me of an example of when this happened and how they initiated this discussion? What was the outcome?
	Do you think stopping a medicine is an easy decision to make? [If not] What makes it difficult to stop a medicine? What do you think makes it difficult for you? Are there any aspects which may facilitate this process?
**Deprescribing: Terminology**	How do you feel about the word deprescribing? Could you suggest an alternative name for it?
**Attitudes, beliefs, expectations about medicines and polypharmacy**	In your view, what are the challenges patients may experience with the daily management of their medicines? How well do you think older patients understand the medicines they have to take?
**Patient-prescriber communication and relationship and decision-making**	How do you ensure patients receive the necessary information about their medicines? Can you give me a recent example of when you ensured this?
	How often do you engage in discussions about your medicines with your patients? Who does normally initiate these discussions? Can you give me a recent example where a patient initiated a discussion about their medicines with you? [Probe] What was the outcome?
	When making decisions about patients' medicines, how do you ensure that patients' priorities are taken into consideration? Can you give me an example of when this happened? How often do patients agree or disagree with a decision you make about their medicines? [If so] Can you talk about it and what the outcome was?
**Final thoughts and considerations**	**Is there any other information, thought or concern you would like to share with me before we end this interview?**
	You have access to systems that help you make decisions about medicines e.g. STOP/START tool, Anticholinergic tool etc. How **do you feel** about using these tools if you do, and what are the **challenges** and **positives** of using them?
**THANK YOU FOR TAKING PART IN THIS STUDY**	

Interview Schedule – Clinicians v.1.3
December 6th, 2017
