# Supplementary material for: Barriers and facilitators of successful deprescribing as described by older patients living with frailty, their informal carers and clinicians: a qualitative interview study
Source: BMJ Open. 2022 Mar 28;12(3):e054279. doi: 10.1136/bmjopen-2021-054279 (PMC8961139; doi:10.1136/bmjopen-2021-054279)
Supplement: Supplementary data [file bmjopen-2021-054279supp003.pdf]

Qualitative interview: Patients (Second interview): v.1.1

Interview Schedule – Patients 2nd interview	
**Topic**	**Questions**
**Deprescribing: Process**	In our last interview we discussed the appointment you had with your healthcare professional where one (or more) medicine(s) was (were) stopped. In these six (to eight) weeks since your appointment, did you experience any issues as a result of stopping this (these) medicine(s)? Can you talk a little bit about your experiences? What strategies did you use to help you overcome these difficulties? What were your concerns?
**Follow-up and on-going monitoring**	Did you seek professional (formal) help at any point? (e.g. GP, practice pharmacist or community pharmacist) If so, can you tell me how it happened? What steps were taken by your healthcare professional to provide you with support? What was the outcome (has it been resolved? How?)
	If (or when) you needed to contact a healthcare professional to discuss your condition and medicines in relation to the medicine(s) that was (were) stopped, were you provided with any contacts you could use? Alternatively: Did you know who to contact if you needed help?
	Were you contacted by your healthcare professional(s) at any point after your medicine(s) was (were) stopped)? If so, who contacted you? Why? How many times? If not, how important would it have been for your healthcare professional to contact you?
	Did you feel you received enough support after your medicine(s) was (were) stopped, or if you did not feel you needed it, do you feel you would have received adequate support if you needed? Who would you contact in the first instance if you needed to ask questions or raise concerns? Why? What was missing?
**Attitudes and behaviours towards deprescribing**	Six (to eight) weeks after you had your medicine(s) stopped, how do you feel? Probes: Would you consider stopping more medicines if your healthcare professional assured this would be good for your health or quality of life? What changed in your opinion comparing to when you stopped this (these) medicine(s) and if you were to stop another medicine in the future?
	What do you think was, or would have been crucial for succeeding in stopping your medicine (what went well and what could have gone better)? What happened that ensured the success? Is there anything else that might need to happen but did not which may have increased the chances of success? Is there anything you would have liked to happen between you and your healthcare professional during this time?
**Final thoughts and considerations**	Is there any other information, thought or concern you would like to share with me before we end this interview?
**THANK YOU FOR TAKING PART IN THIS STUDY**	

Interview Schedule – patients (2nd interview) v.1.1
December 6th, 2017
