# Supplementary material for: Barriers and facilitators of successful deprescribing as described by older patients living with frailty, their informal carers and clinicians: a qualitative interview study
Source: BMJ Open. 2022 Mar 28;12(3):e054279. doi: 10.1136/bmjopen-2021-054279 (PMC8961139; doi:10.1136/bmjopen-2021-054279)
Supplement: Supplementary data [file bmjopen-2021-054279supp004.pdf]

Qualitative interview: Informal carers (First interview): v.1.1

Qualitative interview: Informal carers. Interview 1	
**Topic**	**Questions**
**Deprescribing: Process**	I know the person you help with their medicines had one/a number of your regular medicines stopped by their HCP recently. Can you please tell me about this? Whose decision was it to begin with? [If healthcare professional] Did you agree with the decision? What are your concerns (if any)? Do you think your concerns are the same or different than those of your relative/friend?
	Did you feel you and your relative/friend received enough information and support from the healthcare professional to make you feel comfortable about stopping these medicines? What went well and what could have gone better (during consultation)?
	Were you given the opportunity to fully discuss your thoughts and explore your options?
	Do you think stopping a medicine is an easy decision to make? [If not] What makes it difficult to stop a medicine from your perspective? What do you think makes it difficult for your HCP? What do you think could be done to help stopping problematic medicines effectively?
**Deprescribing: Terminology**	Have you ever heard of the term deprescribing? Do you agree with the term? What comes to mind when you hear the word deprescribing? [Probe] what are your concerns? Would you rather call it something else? [If so] why? What makes you feel that way?
	What would you call the process of stopping medicines?
**Attitudes, beliefs, expectations about medicines and polypharmacy**	What are the challenges you and your relative/friend experience with his/her medicines? How well do you think you think your friend/relative understands the medicines he/she takes? What strategies do you use to ensure your friend/relative takes his/her medicines correctly?
**Patient-prescriber communication and relationship and decision-making**	Do you feel that you are involved in discussions and decisions about your friend/relative's medicines or do you usually defer decisions to the healthcare professional(s)? [if deferral] Why do you think this happens? And are you happy that decisions are deferred to the HCP?
	When making decisions about your relative/friend's medicines, do you often talk to your HCP about yours and your relative/friend's personal care goals and priorities, particularly if you disagree with a decision? Does the HCP ask what your views are on the subject before making a decision? Have you ever disagreed with a decision made by your HCP about your medicines? [If so] Can you talk about it and what the outcome was?
	Do you feel supported and listened to by your healthcare professional(s) when you have questions or concerns about your medicines? [If not] Why not? Can you give me a recent example that made you feel this way?
	Do you think your care goals and priorities for your relative/friend are the same as those of his/hers? If not, how do they differ?
**Final thoughts and considerations**	**Is there any other information, thought or concern you would like to share with me before we end this interview?**
**THANK YOU FOR TAKING PART IN THIS STUDY**	

Interview Schedule – Informal Carers (1st Interview) v.1.1
December 6th, 2017
