# Supplementary material for: Barriers and facilitators of successful deprescribing as described by older patients living with frailty, their informal carers and clinicians: a qualitative interview study
Source: BMJ Open. 2022 Mar 28;12(3):e054279. doi: 10.1136/bmjopen-2021-054279 (PMC8961139; doi:10.1136/bmjopen-2021-054279)
Supplement: Author's manuscript [file bmjopen-2021-054279.draft_revisions.pdf]

BMJ Open is committed to open peer review. As part of this commitment we make the peer review history of every article we publish publicly available.

When an article is published we post the peer reviewers' comments and the authors' responses online. We also post the versions of the paper that were used during peer review. These are the versions that the peer review comments apply to.

The versions of the paper that follow are the versions that were submitted during the peer review process. They are not the versions of record or the final published versions. They should not be cited or distributed as the published version of this manuscript.

BMJ Open is an open access journal and the full, final, typeset and author-corrected version of record of the manuscript is available on our site with no access controls, subscription charges or pay-per-view fees (http://bmjopen.bmj.com).

If you have any questions on BMJ Open's open peer review process please email info.bmjopen@bmj.com

Barriers and facilitators of successful deprescribing as described by older patients living with frailty, their informal carers, and clinicians. A qualitative interview study.

Journal:	*BMJ Open*
Manuscript ID	bmjopen-2021-054279
Article Type:	Original research
Date Submitted by the Author:	23-Jun-2021

Complete List of Authors:	Peat, George; University of Bradford, School of Health and Life Sciences<br>Fylan, Beth; University of Bradford, School of Pharmacy and Medical Sciences; Bradford Institute for Health Research Yorkshire Quality and Safety Research Group<br>Marques, Iuri; University of Bradford, School of Pharmacy and Medical Sciences<br>Raynor, David; University of Leeds, School of Healthcare<br>Breen, Liz; University of Bradford, School of Pharmacy and Medical Sciences<br>Alldred, David; University of Leeds, School of Healthcare<br>Olaniyan, Janice; University of Bradford, School of Health and Life Sciences

Keywords:	PRIMARY CARE, QUALITATIVE RESEARCH, GERIATRIC MEDICINE

SCHOLARONE™
Manuscripts

I, the Submitting Author has the right to grant and does grant on behalf of all authors of the Work (as defined in the below author licence), an exclusive licence and/or a non-exclusive licence for contributions from authors who are: i) UK Crown employees; ii) where BMJ has agreed a CC-BY licence shall apply, and/or iii) in accordance with the terms applicable for US Federal Government officers or employees acting as part of their official duties; on a worldwide, perpetual, irrevocable, royalty-free basis to BMJ Publishing Group Ltd ("BMJ") its licensees and where the relevant Journal is co-owned by BMJ to the co-owners of the Journal, to publish the Work in this journal and any other BMJ products and to exploit all rights, as set out in our licence.

The Submitting Author accepts and understands that any supply made under these terms is made by BMJ to the Submitting Author unless you are acting as an employee on behalf of your employer or a postgraduate student of an affiliated institution which is paying any applicable article publishing charge ("APC") for Open Access articles. Where the Submitting Author wishes to make the Work available on an Open Access basis (and intends to pay the relevant APC), the terms of reuse of such Open Access shall be governed by a Creative Commons licence – details of these licences and which Creative Commons licence will apply to this Work are set out in our licence referred to above.

Other than as permitted in any relevant BMJ Author's Self Archiving Policies, I confirm this Work has not been accepted for publication elsewhere, is not being considered for publication elsewhere and does not duplicate material already published. I confirm all authors consent to publication of this Work and authorise the granting of this licence.

For peer review only

Barriers and facilitators of successful deprescribing as described by older patients living with frailty, their informal carers, and clinicians. A qualitative interview study.

Authors: George Peat [1,2,4], Beth Fylan [1,2,4,5], Iuri Marques[1,2,4], David K Raynor [2,3], Liz Breen [1,2,4], David Alldred [2,3,4], Janice Olaniyan [1,2,4].

*Corresponding author: George Peat ORCID 0000-0002-0293-2456. Email: gwpeat@bradford.ac.uk. Address: George Peat, School of Pharmacy and Medical Sciences, University of Bradford, Bradford, BD7 1DP, UK.

1. School of Pharmacy and Medical Sciences, University of Bradford, Bradford, UK.

2. NIHR Yorkshire and Humber Patient Safety Translational Research Centre (YH PSTRC), Bradford, UK.

3. School of Healthcare, University of Leeds, Leeds, UK.

4. Wolfson Centre for Applied Health Research, Bradford, UK

5. Yorkshire Quality and Safety Research Group, Bradford Institute for Health Research, Bradford, UK.

Key words: Primary care, Qualitative research, Geriatric medicine

Word count: 4221

For peer review only

TITLE: Barriers and facilitators of successful deprescribing as described by older patients living with frailty, their informal carers, and clinicians. A qualitative interview study.

AUTHORS:

Peat G, Fylan B, Marques I, Raynor DK, Breen L, Alldred D P, Olaniyan J.

ABSTRACT

Aim/Objective

To explore the barriers/facilitators to deprescribing in primary care in England from the perspectives of clinicians, patients living with frailty who reside at home, and their informal carers, drawing on the Theoretical Domains Framework (TDF) to identify behavioural components associated with barriers/facilitators of the process.

Design

Qualitative semi-structured interviews took place with patients living with frailty, their informal carers, and clinicians. Patients (n=9) and informal carers (n=3) were interviewed twice: immediately after deprescribing, and five/six weeks later. Clinicians (n=14) were interviewed once (n=38 interviews). All interviews were audio-recorded and transcribed verbatim. Framework analysis was applied to analyse the data. Two broad themes representative of the barriers and facilitators to deprescribing experienced by participants were first identified. Each broad theme was explored to identify sub-themes representative of a barrier or facilitator of deprescribing. Finally, each sub-theme was mapped to the TDF to identify domains to target for deprescribing interventions.

Results

Six themes associated with facilitators and barriers to deprescribing were generated respectively, with each supported by between two and three sub-themes. Themes representative of facilitators of deprescribing were most frequently mapped onto the Skills, Knowledge, and Goals domains of the TDF. Themes representative of the barriers of deprescribing were most frequently mapped onto the environmental context and resources, memory, attention, and decision processes, and emotion domains of the TDF.

Conclusion

This paper offers timely insight into the barriers and facilitators to deprescribing within the context of primary care in England. As deprescribing continues to grow in national and international significance, it is important that future deprescribing interventions acknowledge the current barriers and facilitators and their associated behavioural components experienced by clinicians, patients living with frailty, and their informal carers to improve the safety and effectiveness of the process.

STRENGTHS AND LIMITATIONS OF THE STUDY

- A strength and novel aspect of the study was the interviewing of patients about their deprescribing journey at two timepoints, immediately after their deprescribing consultation and 4 to 6 weeks later.
- A strength of the study was the inclusion of patients, their informal carers, and healthcare professionals collectively, providing a multi-perspective insight into the barriers and facilitators of deprescribing in primary care in England.
- Our sample of patients and informal carers was lower than expected, due to challenges recruiting patients who were actively having a medicine deprescribed.

For peer review only - http://bmjopen.bmj.com/site/about/guidelines.xhtml

INTRODUCTION

Frailty can be understood as the age-related decline in physiological structures that can result in, among other consequences, an increased vulnerability to adverse drug reactions (ADRs). This is due to the interplay between physiological changes and pharmacokinetics and pharmacodynamics.(1) Consequently, there is an increasing focus on the safe and appropriate use of medicines in patients living with frailty. One strategy to improve medicines safety is to reduce or stop medicines that may no longer be appropriate or beneficial, a process referred to as deprescribing.(2)

If undertaken appropriately, deprescribing has been shown to support a reduction in ADRs and can improve quality of life.(3) Several frameworks for deprescribing exist (e.g. 2;4-6), however there is uncertainty regarding their effectiveness. This means that identifying factors that facilitate or hinder deprescribing continues to be a high priority to ensure the process of deprescribing is safely and effectively implemented to optimise patient outcomes.(7)

Evidence suggests that barriers to deprescribing exist at various socio-ecological levels.(7) For instance, several studies have found clinicians in primary care face time restraints that hinder effective deprescribing, including limited time to review medicines, engage in consultations, and provide adequate follow-up.(8-10) Interpersonal barriers also exist and include ineffective communication with prescribers across healthcare settings, with some prescribers hesitant to deprescribe medicines prescribed by specialists in other care settings.(9, 11, 12)Challenges with communicating deprescribing effectively to patients using appropriate discourse exist.(11) Similarly, studies have reported prescriber hesitancy to deprescribe due to limited knowledge and guidance on how to do so appropriately and safely.(8; 13, 14) Finally, clinician hesitancy to deprescribe also exists because of a fear that deprescribing may be perceived by the patient as a withdrawal of care.(8)

From the perspective of patients living with frailty, managing multiple morbidities often means engaging with multiple prescribers over a sustained period, with evidence suggesting this scenario can make deprescribing challenging. For instance, an increased risk of poor communication between parties involved in a patient's care has been cited, resulting in patients confused about which prescriber has authority to deprescribe.(9) Similarly, patients prescribed medicines for a significant period are often reluctant to stop those medicines, particularly if they believe that the medicine is necessary, or that stopping the medicine would result in negative outcomes.(8;15) Likewise, older adults often lack sufficient knowledge about their medicines, limiting confidence and willingness to engage in decision making related to deprescribing.(14) Finally, paternalistic prescribing attitudes, void of shared decision-making, have also been shown to be a barrier to deprescribing.(14,15)

Studies have also reported facilitators of deprescribing. These include ensuring clinicians receive sufficient information and support related to the risks and benefits of deprescribing specific medicines to enable an informed decision.(13) Similarly, adopting an organisational culture, which encourages peer-to-peer learning, has been found to improve the self-efficacy of clinicians to deprescribe.(11,17) From a patient perspective, factors such as shared decision-making, trust in the clinician as a result of familiarity built through sustained clinician-patient relations, and a desire to reduce medicines have been found to support deprescribing.(9, 18,19)

Despite a growing evidence-base on barriers to and facilitators of deprescribing, studies largely exist outside of the UK with only three out of forty studies identified in a recent systematic review on deprescribing in primary care.(7) More so, there is a paucity in research into deprescribing focused specifically on patients living with frailty, a patient cohort

For peer review only - http://bmjopen.bmj.com/site/about/guidelines.xhtml

increasingly considered a priority group for interventions that improve medicines safety. For example, recent policy in England, such as the General Medical Services (GMS) contract, requires General Practice (GP) to conduct Structured Medication Reviews (SMR) with patients living with moderate to severe frailty.(20) A potential outcome of an SMR is that a patient may have one or more of their medicines stopped. To support the success and effectiveness of deprescribing as a potential outcome of SMRs for patients living with frailty, clinicians need to be aware of the barriers to and facilitators of deprescribing in this group. Furthermore, while international studies have aimed to understand the patient perspective on deprescribing, no studies appear to have captured the barriers/facilitators of deprescribing from the perspective of patients who were actively having a medicine reduced or stopped. Finally, few studies have identified the determinants of behaviour that either facilitate, or act as a barrier to deprescribing. To address these gaps, this study aimed to explore the barriers/facilitators to deprescribing from the perspectives of clinicians, patients living with frailty who reside at home and were candidates for deprescribing, and their informal carers in the specific context of primary care in England, drawing on the Theoretical Domains Framework (TDF) to identify behavioural components associated with barriers/facilitators of the process.

METHODS

Study design, setting, and sampling (inclusion/exclusion)

The study adopted a qualitative design and recruited from four GP practices across regions of Yorkshire and Humber in England. Purposeful sampling was used to recruit patients ≥ 65 years old, living with frailty (either formally diagnosed and registered in the primary care electronic health record or at risk of frailty as defined by the electronic Frailty Index, eFI embedded within GP prescribing systems 21) who attended a consultation to deprescribe. In addition, informal carers who provided informal care to patient participants were also recruited to the study. Clinicians recruited eligible patients if during their appointment a medicine had been identified for deprescribing. Patients were provided an invitation letter and participant information sheet. Those who agreed to participate gave signed, informed consent. Patients were asked if they had an informal carer and invitations were extended to them.

To capture the perspective of clinicians, healthcare professionals involved in deprescribing (GPs, pharmacists, nurses and pharmacy technicians) were interviewed. Clinicians were recruited from the four participating surgeries. They were approached by a practice representative and were invited to take part, received an invitation letter and participant information sheet. Those who agreed to participate gave signed, informed consent. All clinicians had experience of deprescribing.

Data collection

Semi-structured interviews were conducted between September 2018 and February 2019. Interviews with clinicians took place at their practice and explored their experiences of deprescribing. Participants provided consent for the interviews to be audio-recorded and transcribed verbatim. Two interview schedules, informed by the literature and the Theoretical Domains Framework (TDF), and developed in consultation with a patient and public involvement and engagement member (PPIE), were designed to capture experiences of deprescribing and potential barriers and facilitators during the process.(22) The TDF, which is a theoretical framework used to understand behavioural influences, was used to focus and facilitate discussions about the determinants of behaviours of clinicians, patients, and their informal carers. Patients and their informal carers were interviewed twice at their homes, up to one week after their appointment, and five-six weeks later. The first interview explored patients' immediate perceptions of deprescribing, while the second focused on outcomes and their experience of the process. Interviews lasted on average 30 minutes. Interviews

For peer review only - http://bmjopen.bmj.com/site/about/guidelines.xhtml

were conducted by experienced healthcare researchers from a range of backgrounds: a psychologist experienced in pharmacy practice and patient safety research, a sociologist and a pharmacist.

Data analysis

Framework analysis was applied to analyse the data.(23) The approach provides a practical and structured approach to data management and analysis through five stages of Framework Analysis: data familiarisation; iterative development of a framework for the purpose of data management; indexing of individual transcript data to the categories within the framework; summarising the data within each category; interpretation of the data.(23)Data was coded by GP, BF, and IM, with LB, DA, DKR, and JO providing feedback on the coding framework. Two broad master themes representative of the barriers and facilitators to deprescribing experienced by participants were first identified. Each broad theme was then explored to identify themes and sub-themes representative of a barrier or facilitator of deprescribing. Finally, each sub-theme was mapped to the TDF to identify domains to target for deprescribing interventions.

Patient and public involvement

All patient-facing recruitment materials, including the topic guide/s, were reviewed by a patient public involvement and engagement (PPIE) representative.

RESULTS

38 interviews in total were undertaken. Nine patients were interviewed at two separate points, up to one week after appointment and five to six weeks later, along with three informal carers (Table 1). Fourteen clinicians were also interviewed once, comprising of 6 GPs, 2 practice pharmacists, 5 practice nurses and advance nurse practitioners, and 1 pharmacy technician (Table 2). Analysis of the data identified three themes associated with facilitators of deprescribing, with each theme supported by between two and three sub-themes (Figure 1). Three themes associated with barriers of deprescribing were also identified and were similarly supported by between two and three sub-themes (Figure 2). Tables 3 and 4 illustrate the themes and sub-themes identified, with each mapped onto the TDF to identify behavioural components associated with barriers and facilitators of deprescribing. Themes and sub-themes associated with facilitators to deprescribing were attributed to the following TDF domains: *Skills, Intentions, Belief about capabilities, Knowledge, Social influences, Social/professional role and identity, Environmental context and resource, Memory, attention, and decision processes, Goals.* Conversely, the themes and sub-themes associated with barriers to deprescribing were attributed to the following domains: *Social influences, Environmental context and resources, Social/professional role and identity, Memory, attention, and decision processes, Skills (interpersonal), Emotion.*

Each whole theme and their sub-themes representative of a barrier, or facilitator of successful deprescribing are summarised in the next section.

For peer review only - http://bmjopen.bmj.com/site/about/guidelines.xhtml

Table 1- Demographics: Patients and informal carers

#	Patient Code	Age	Gender	Ethnicity	Number of medicines before deprescribing	Known diagnoses (patient reported)	Informal carers	
							Code	Relationship
**1**	Practice 1-Patient1	Early 70s	Male	White British	5	Diabetes, Hypertension	N/a	
**2**	Practice 1-Patient 2	Late 80s	Male	White British	15	COPD, Rhinitis, Arthritis, Angina, Dry Eyes	N/a	
**3**	Practice 1—Patient 3	Late 80s	Male	White British	8	Heart valve replacement, pacemaker	N/a	
**4**	Practice 2-Patient 1	Early 90s	Male	White British	7	Diabetes, Hypertension, Chronic diverticulitis, Hiatus Hernia, Kidney strain (borderline failure)	N/a	
**5**	Practice 2 - Patient 2	Mid 80s	Male	White British	5	Diabetes, Hypertension	N/a	
**6**	Practice 3 - Patient 2	Early 70s	Female	White British	15	Restless legs syndrome, generalised pain, early-stages dementia, constipation	N/a	
**7**	Practice 3 - Patient 3	Late 70s	Female	White British	9	Type 2 diabetes, Osteo/Rheumatoid Arthritis, high blood pressure, high cholesterol, has had mini stroke, possible cataracts or glaucoma	Practice 3 -IC3	Daughter
**8**	Practice 4 - Patient 1	Late 70s	Female	White British	16	Pacemaker, type 2 diabetes, thyroid, hiatus hernia, lymphedema, high blood pressure	Practice 4-IC1	Daughter-in-law
**9**	Practice 4 - Patient 3	Late 70s	Male	White British	14	Arthritis, diabetes, dormant leukaemia	Practice 4-IC3	Wife

For peer review only - http://bmjopen.bmj.com/site/about/guidelines.xhtml

Table 2- Demographics: Clinicians

#	HCP Code	Gender	Ethnicity	Job Role	Prescriber	Years and Type of Experience
1	Practice 1-HCP1	Female	White British	GP	Yes	15 years as a doctor, 8 years as a GP
2	Practice 1-HCP2	Female	White British	Practice Pharmacist	No	34 years as a pharmacist, 9 months in primary care
3	Practice 1-HCP3	Female	White British	Practice Nurse	Yes	23 years as a nurse, 14 years in primary care, 15 months as prescriber
4	Practice 2-HCP1	Female	White British	GP	Yes	25 years as a doctor, 22 years as a GP
5	Practice 2-HCP2	Female	White British	Pharmacy Technician	No	32 Years as pharmacy technician, 6 years in primary care
6	Practice 2-HCP3	Female	White British	Advanced Nurse Practitioner	Yes	20 years as a nurse, 15 years in primary care, 3 years as prescriber
7	Practice 3-HCP1	Female	White British	Advanced Nurse Practitioner	No	44 years as a nurse, 10 years in primary care
8	Practice 3-HCP2	Female	White British	GP	Yes	28 years as a doctor, 23 years as a GP
9	Practice 3-HCP3	Male	White British	GP	Yes	11 years as a doctor, 6 years as GP
10	Practice 3-HCP4	Female	White British	GP	Yes	20 years as a doctor, 14 years as GP
11	Practice 4-HCP1	Female	White British	GP	Yes	7 years as a doctor, 1,5 years as a GP
12	Practice 4-GCP2	Female	White British	Practice Nurse	Yes	29 years as a nurse, 12 years as practice Nurse, 8 years as a prescriber
13	Practice 4-HCP3	Female	White British	Practice Nurse	Yes	4 years as a nurse, 2 years in primary care and as prescriber
14	Practice 4-HCP4	Female	White British	Practice Pharmacist	No	13 years as a pharmacist, 2,5 years in primary care

For peer review only - http://bmjopen.bmj.com/site/about/guidelines.xhtml

Table 3- Facilitators of deprescribing

Table 3. Facilitators of deprescribing			
**Theme**	**Sub-theme**	**TDF domain**	**Sample quotes**
**Deciding to deprescribe**	Treatment burden	Intentions	"I'm glad those things have reduced". P2 (Practice 1)<br>"We get quite a few people who are on a lot of medication, don't generally know why they take certain ones. And you do get a lot of them saying is there any of these that I could actually stop?" HCP2 (Practice 4).
	Shared decisions	Belief about capabilities<br>Skills (ability)	"I am a great believer in shared decision making so I do spend a lot of my consultations around making sure that [the patient knows the reason for taking the medicine], because their concordance (adherence) will be very poor if they don't understand why they are taking it and they need to understand the importance" HCP3 (Practice 1).
	Information and communication	Skills (interpersonal)<br>Knowledge (procedural)	"…you're doing something that they ultimately disagree with and you're telling them that they can't have something that they want, that's the crux of the matter, but you also have to use your communication skills to explain why you're doing that, why is it that you're stopping this medication." HCP3 (Practice 3).
**Relationships**	Trust in clinicians	Skills (interpersonal)	"I think knowing the patient, so if I've met the patient before that always makes the process easier, so I'll have already had a little bit of rapport with them." HCP3 (Practice 3)<br>"So, I think for this lady I knew her, so I had a relationship with her which helps. And I think building that understanding and trust between us, so I think it was positive that we had trust…" HCP4 (Practice 4)
	Role of informal carers	Social Influences (social support)	"She can't always take in what's being said. If I had known that they were going to have her in to take her medication away I'd have gone with her, so that I could listen to the explanations that were being given to her to allay her worries." Supporting peer 1 (practice 4

For peer review only - http://bmjopen.bmj.com/site/about/guidelines.xhtml

Table 3. Facilitators of deprescribing (cont)

Theme	Sub-theme	TDF domain	Sample quotes
**Relationships (cont)**	Multidisciplinary working	Social/professional role and identity (organisational commitment)<br>Environmental context and resource (organisational culture)	“We’ve done a lot of deprescribing work here and it has been successful because we decided to involve the whole team. It would be no good if I sat here in a practice with six GPs and decided to improve my deprescribing because the other five GPs would be quietly sabotaging that without realising because we’re not working together. If we all change our deprescribing patterns, then we have a hope of really making [a] change” HCP1 (Practice 1)<br><br>“Probably experience of doing the same thing for other patients, so if it’s a drug that we’re reviewing all of our patients on and having discussions about continuing them, if I’ve already had a similar conversation with someone previously that would make the process easier for me, so experience really" HCP3 (Practice 1)<br><br>"Similarly I’ve brought in the specialist, I’d already got the agreement of the GP involved in the patient’s care, this was about course of action and it was highlighted to the healthcare assistant who she normally sees as well that it was you know the incorrect treatment for the patient."<br>I: "What was the outcome?"<br>R: "She has now accepted that it’s not the right course of treatment for her and it can actually cause more difficulties" HCP3 (Practice 3)

For peer review only - http://bmjopen.bmj.com/site/about/guidelines.xhtml

Table 3. Facilitators of deprescribing (cont)			
**Theme**	**Sub-theme**	**TDF domain**	**Sample quotes**
**Process of deprescribing**	Gradual introduction	Skills (interpersonal)<br>Memory, attention, and decision processes<br>Reinforcement<br>Beliefs about consequences<br>Goals<br>Skills (competence)	"The way she talked to me and told me about it because she says don't think anything is going to happen because it isn't...She said you're lowering your dosage that's all..." Patient 2 (Practice 3).<br><br>"I think as long as you kind of go you know 'Obviously if you ever do need it again of course we'll restart it again you know, but let's just do it really slowly and we'll do it together and I'll keep an eye on you and you'll come and see me and we'll review you' I think most people are actually quite happy to at least give it a go and try and come off medication" HCP1 (Practice 2)<br><br>"It was about a control thing, they start off with one thing, one little step, and then they came back and said how they felt about that step; for this case it was pregabalin, and I talked about how to reduce it and if actually they got side effects or if things got worse it was then to speak and actually go further; just wait a little while and then make your next step, and giving them back some of the control, so they felt in charge rather than me, and that always seems to work much better" HCP2 (Practice 3)<br><br>We sort of went backwards and we discussed about why it had initially been started and what it did for her and how that worked. And then explained about how the symptoms linked in with how it was working, and it was working too well. So, sort of empowering her to make that connection as I had and then we both then came to the same point of "we need to stop it" HCP4 (practice 4)

For peer review only - http://bmjopen.bmj.com/site/about/guidelines.xhtml

Table 3. Facilitators of deprescribing (cont)			
**Theme**	**Sub-theme**	**TDF domain**	**Sample quotes**
**Process of deprescribing (cont)**	Support, monitoring, follow up	Goals (target setting)<br>Knowledge (procedural)	On that occasion [I received] as much [support] as I'd wanted, previously I'd discussed it [discontinuing Aspirin] with [my GP] and with other practitioners at the health centre and been very, felt very supported" Patient 1 (Practice 1)<br><br>Interviewer: "Do you feel you have been receiving enough support after your medicine was stopped or tapered?"<br>Respondent: "Yes, I agreed with [clinician] we agreed three months would be ideal for her to review me and see what's going on." (Respondent code here. E.g. Patient x, Practice x)<br><br>“She (diabetic nurse) always tells me if there's anything either worse or better all that I can always get in touch with her if needs be”. P3 (Practice 3).

For peer review only - http://bmjopen.bmj.com/site/about/guidelines.xhtml

Table 4: Barriers to deprescribing

Table 4- Barriers to deprescribing			
**Theme**	**Sub-theme**	**TDF domain**	**Sample quotes**
**Organisation of healthcare**	Clinician continuity	Social influences<br>Environmental context and resources	"You can't get in touch with your Doctor. It's no good going to another Doctor that doesn't understand that you're coming off your morphine and how it's affecting you." Patient 2 (Practice 3)<br><br>"You never see the same doctor twice (...) unless you're under one person all the time you could go haywire (...) That's often the way when you see one doctor after another, you, frequent doctor gets changed. They jump in too quick, if you're under the care of one person then that person can really monitor you" Patient 1 (Practice 2)
	Consultation constraints	Environmental context and resources	"We're in tough times in primary care and pressure of demand is really, really high and so ad-hoc deprescribing is really difficult to do" HCP1 (Practice 1).<br>"I think it might have been easier if I hadn't done it [reviewed patient's medicines] over the phone, it might have actually been easier with that patient in front of me, but I've got time limitations" HCP2 (Practice 1)<br>"They haven't got time you see; you go into the surgery and you've only 10 minutes" Carer 3 (Practice 4)
	Perceived hierarchies	Social/professional role and identity	"I can't remember the exact drug, but I can tell you it was a cardiology medication, and the resistance really was they'd been told, you know this Cardiologist had told them they needed it and they needed to have it lifelong." HCP1(Practice 4)

For peer review only

For peer review only - http://bmjopen.bmj.com/site/about/guidelines.xhtml

**Table 4. Barriers to Deprescribing (cont.)**			
**Theme**	**Sub-theme**	**TDF domain**	**Sample quotes**
**Communication clarity**	Accessibility of terminology and information	Memory, attention, and decision processes	"I think [patients] get a bit confused by the word deprescribing. It can be very much a clinical term. we need to accept that patients aren't [always familiar with clinical terms] and so I would quite often call it something different" HCP2 (Practice 2)<br><br>"My first impression was that [deprescribing] was something that was done to one not something that you had an active say in. Yeah but it was a decision that a healthcare professional took and told the patient about it" Patient 1 (Practice 1)
	Sharing and understanding decisions	Memory, attention, and decision processes<br>Skills (interpersonal)	"I don't want them to say right we'll start reducing this, I need to know why and what's going to happen with me. " Patient 2 (Practice 3)<br>"They don't explain what they're trying to say to you. It's as if oh we'll see you next week, go on we'll see you again later. It's…not explained what they're telling you…" Patient 3 (Practice 4).
**Emotional factors**	Medicines-taking habit	Emotion	"Because she [patient] panics. And she's been on them such long-term and it becomes habit. And I think when you stop something that you've been doing for a long time you think 'oh', you panic straight away, you think 'oh, it's all going to go wrong, it's all going to go wrong" Carer 1 (Practice 4).
	Fear of negative outcomes	Emotion	"Yes, I was afraid. Because the morphine helped me with the pain and I knew each time I was coming down I was getting pain, so I just took paracetamol and then it just kept coming down I think it was about every month she wanted me to have a go at coming down." (GP3-P2).

For peer review only - http://bmjopen.bmj.com/site/about/guidelines.xhtml

For peer review only

14

Insert Figure 1

Insert Figure 2

Facilitators to Deprescribing

Theme One: Deciding to Deprescribe

Arriving at a decision to deprescribe a medicine(s) was facilitated by several factors. First, some patients wished to reduce their medicines, a viewpoint reflected both in the accounts of patients and clinicians (Table 3). To reach a deprescribing decision, clinicians also advocated shared decision-making during consultation:

> *"I am a great believer in shared decision making so I do spend a lot of my consultations around making sure that [the patient knows the reason for taking the medicine], because their concordance (adherence) will be very poor if they don't understand why they are taking it…" HCP3 (Practice 1).*

Ensuring a patient understood why deprescribing was appropriate was essential in maintaining adherence with a new regimen, with shared decision-making integral to developing this understanding. Similarly, effectively communicating why deprescribing was appropriate was important, particularly with patients who were initially hesitant to deprescribe (Table 3):

Theme two: Relationships

Positive relationships between involved parties were important to facilitating deprescribing. Established trust, built through a knowingness between patient and clinician was highlighted as a facilitator by participants (Table 3). In addition, involving informal carers in deprescribing consultations was cited as beneficial to assist patients where necessary to understand why their medicine/s were deprescribed. (Table 3). Furthermore, the relational dynamic between clinicians and their practice was cited as significant. For instance, participants described the benefits of adopting a practice agenda to deprescribing:

"We've done a lot of deprescribing work here and it has been successful because we decided to involve the whole team. It would be no good if I sat here in a practice with six GPs and decided to improve my deprescribing because the other five GPs would be quietly sabotaging that without realising because we're not working together. If we all change our deprescribing patterns, then we have a hope of really making [a] change" HCP1 (Practice 1)

Care for older people living with frailty often involves multiple clinicians and healthcare workers. Working across teams to inform those involved in the patient's care of the decision to deprescribe was important to supporting the patient to accept their new medicines regimen:

Participant: "I've brought in the specialist, I'd already got the agreement of the GP involved in the patient's care, this was about course of action and it was highlighted to the healthcare assistant who she normally sees as well that it was you know the incorrect treatment for the patient." HCP3 (Practice 3)

Interviewer: "What was the outcome?"
Participant: "She has now accepted that it's not the right course of treatment for her and it can actually cause more difficulties" HCP3 (Practice 3)

For peer review only - http://bmjopen.bmj.com/site/about/guidelines.xhtml

Theme three: Process of deprescribing

Treating deprescribing as a process, as opposed to a one-off event, was a key facilitator relayed by participants. Patients described feeling more assured and confident of the process if it was conveyed as a gradual reduction in their medicine(s). Gradually reducing dosage of medicines was associated with having a sense of control by some participants, indeed, clinicians expressed the importance of allowing the patient 'to feel in charge' of the process:

> *"It was about a control thing, they start off with one thing, one little step, and then they came back and said how they felt about that step; for this case it was pregabalin, and I talked about how to reduce it and if actually they got side effects or if things got worse it was then to speak and actually go further; just wait a little while and then make your next step, and giving them back some of the control, so they felt in charge rather than me, and that always seems to work much better" HCP2 (Practice 3)*

Deprescribing was described as 'much better' when patients were enabled to feel in control, a feeling that manifested from ensuring the patient was informed, and that steps to reduce their medicines were made tentatively in a controlled fashion:

> *"I think as long as you kind of go you know 'Obviously if you ever do need it again of course we'll restart it again you know, but let's just do it really slowly and we'll do it together and I'll keep an eye on you and you'll come and see me and we'll review you' I think most people are actually quite happy to at least give it a go and try and come off medication" HCP1 (Practice 2)*

Barriers

Theme one: Organisation of healthcare

Aspects of the structure and organisation of healthcare were perceived as barriers to deprescribing. Participants described difficulty in seeing the same clinician, without clinician continuity, some participants felt that they were unable to be sufficiently monitored, and their deprescribing journey would not be understood:

> *"You never see the same doctor twice... unless you're under one person all the time you could go haywire [frustrated]... That's often the way when you see one doctor after another. They jump in too quick. If you're under the care of one person then that person can really monitor you" Patient 1 (Practice 2)*

Time limitations associated with the consultation were a further barrier. Clinicians described how time demands placed on primary care did not facilitate ad-hoc deprescribing. Similarly, time limitations influenced the type of deprescribing consultations clinicians offered. For example, one participant expressed that while face-to-face consultations were 'easier,' due to time limitations, reviews often took place over the telephone:

> *"I think it might have been easier if I hadn't done it [reviewed patient's medicines] over the phone, it might have actually been easier with that patient in front of me, but I've got time limitations" HCP2 (Practice 1)*

Time limitations on consultations were also voiced by carer participants, with the perception that clinicians 'haven't got the time':

"They haven't got time you see; you go into the surgery and you've only 10 minutes" Carer 3 (Practice 4)

For peer review only - http://bmjopen.bmj.com/site/about/guidelines.xhtml

For peer review only

The perceived hierarchy associated with clinicians was also voiced as a barrier to deprescribing. Participants spoke of this being a particular issue when patients had been prescribed medicines by a specialist in the past, who may have conveyed that the medicine(s) were a 'lifelong' regimen:

> *"I can't remember the exact drug, but I can tell you it was a cardiology medication, and the resistance really was they'd been told, you know this Cardiologist had told them they needed it and they needed to have it lifelong." HCP1 (Practice 4)*

Theme two: Communication clarity

Challenges to communicating deprescribing effectively were identified as barriers to deprescribing. First, the term 'deprescribing' was thought to be a 'clinical term' and therefore created an impression that it '*was something that was done to one, not something you had an active say in.' (Patient 1, Practice 1).* Clinicians also felt patients may feel *'a bit confused by the word deprescribing.' (HCP2, Practice 2).* More so, terminology was a particular barrier in settings where the practice had *'quite a number of different populations with lots of different languages' (HCP3, Practice 2).*

Communication barriers also related to the communicative make-up of the deprescribing consultation. For example, some patients felt that clinicians did not spend the time explaining why the decision to deprescribe may have been appropriate:

> *"They don't explain what they're trying to say to you. It's as if oh we'll see you next week. It's…not explained what they're telling you…" Patient 3 (Practice 4).*

The importance of ensuring communication clarity in relation to the topic of deprescribing, particularly in the case of older people living with frailty was voiced by one clinician:

> *"I think sometimes people, if they're slightly older, can worry that they're just sort of, being written off".* HCP2 (Practice 4)

Theme three: Emotional factors

The emotional factors associated with deprescribing were most pertinent when participants discussed the habitual nature of a patient's medicines regimen, and fears of possible negative outcomes from stopping medicines. For example, clinicians described how *"Some people don't like change, there's a resistance to change for some patients" (HCP3, Practice 1).* The habitual connection to medicines also caused some patients to 'panic' when such medicines were tapered or stopped:

> *"Because she [patient] panics. And she's been on them such long-term and it becomes habit. And I think when you stop something that you've been doing for a long time you think 'oh', you panic straight away, you think 'oh, it's all going to go wrong, it's all going to go wrong" Carer 1 (Practice 4).*

For peer review only - http://bmjopen.bmj.com/site/about/guidelines.xhtml

DISCUSSION

Several key findings were identified that contribute to the existing evidence base on deprescribing for patients living with frailty. First, and in relation to the TDF domains *environmental context and resources* and *social/professional role and identity*, adopting deprescribing as a whole practice/team agenda whereby resource is directed towards deprescribing is likely to lead to better outcomes. More so, encouraging multidisciplinary working appears to be particularly important to successfully managing deprescribing for patients living with frailty, who often have multiple morbidities managed by multiple clinicians. Ensuring all clinicians involved in the patient's care were aware of the decision to deprescribe, was found to be important in supporting the patient to accept their new medicines regimen. *Environmental and resource factors* were also found to hinder deprescribing efforts. Patients voiced frustrations related to the inability to see the same clinician. Furthermore, while instances of successful deprescribing consultations and follow-up were reported, so too were instances of patients feeling that they did not have the time to discuss the decision to deprescribe, with some patients also feeling dissatisfied about the follow-up care offered.

A key facilitator of deprescribing identified was the attribution of control to the patient throughout the deprescribing process. In relation to the TDF, domains related to *skills (interpersonal), goals*, and *belief about consequences*, were identified. For example, interpersonal skills were necessary to convey an attribution of control to the patient. Likewise, where clinicians, patients, and informal carers were able to agree on specific and gradual goals, patients' beliefs about the consequences of deprescribing were well managed. Furthermore, while some patients living with frailty may need to be supported by informal carers throughout the process, our findings suggest that all patients benefit from feeling informed, engaged, and in control of the process of deprescribing. Identifying the TDF domains associated with barriers or facilitators of deprescribing, will help focus future intervention development aimed at improving deprescribing outcomes in elderly patients living with frailty. Specifically, future interventions should target behaviour domains linked to *environmental context and resources, social/professional role and identity, and skills (interpersonal).*

Several limitations are also acknowledged. First, the number of patients and informal carers interviewed was lower than expected. Recruitment was challenging in relation to timing recruitment to when patients were having a medicine deprescribed. Similarly, the study could have benefitted from the inclusion of more informal carers. These limitations notwithstanding, the data provided valuable insight into the real-time barriers and facilitators experienced by clinicians, patients, and their informal carers. Furthermore, a major strength, and novel aspect of this qualitative study was the fact that we interviewed patients at two-time points whilst they were *actually* having a medicine deprescribed. Interviewing patients immediately after consultation and six weeks later, provided useful insight into their experience of follow-up, highlighting practices that facilitate or hinder deprescribing.

NHS England's implementation guidance for Structured Medication Reviews (SMRs) states the importance of shared decision-making and emphasises the SMR as an ongoing process as opposed to a one-off exercise.(24) Our findings support previous international evidence that highlights that patients over the age of 65 living with frailty want to be involved in decisions related to deprescribing.(18) Patients living with frailty are a priority cohort for SMRs, consequently, it is important that implementation guidance is acted on, particularly regarding ensuring patients are engaged in any deprescribing decisions made. Furthermore, while it is the case that patients may be unwilling to deprescribe if they believe their medicine is appropriate,(15) this study found patients living with frailty were keen to engage in discussions about their medicines and how to reduce them, a finding shared elsewhere in

For peer review only - http://bmjopen.bmj.com/site/about/guidelines.xhtml

the literature.(18) Consequently, these findings indicate that patients may be generally more open to the prospect of deprescribing than perceived.

Acknowledging the barriers to deprescribing as experienced by clinicians, patients living with frailty, and their informal carers is integral to improving the effectiveness and acceptability of the process. The term 'deprescribing' was perceived by patients as either confusing, or suggestive of a process led by the clinician as opposed to being shared. Similar concerns with deprescribing discourse have also been voiced in the literature(18) suggestive of a requirement to rethink how the process is conveyed to patients and the public. To successfully deprescribe, the views and concerns of patients need to be discussed as part of the consultation.(5,15, 25) Several participants voiced concerns about not understanding why their medicines were being stopped, a finding underreported in the current evidence base. The shared commonalities in reported barriers/facilitators of deprescribing reported by this study and the wider literature highlight components of the process that future deprescribing intervention studies should seek to address, and those that should be prioritised to improve deprescribing effectiveness. Furthermore, identifying the barriers and facilitators of deprescribing and their associated behavioural domains, can support clinicians to deprescribe safely and effectively as part of the NHS mandated Structured Medicine Review programme.

In conclusion, this paper offers a timely contribution to the existing evidence base, providing insight into the barriers and facilitators to deprescribing experienced by clinicians, patients living with frailty and their informal carers within the context of primary care in England. As deprescribing continues to grow in national and international significance, it is important that future deprescribing interventions acknowledge the current barriers and facilitators and their associated behavioural components faced by clinicians, patients living with frailty, and their informal carers to improve the safety and effectiveness of the process.

For peer review only

For peer review only - http://bmjopen.bmj.com/site/about/guidelines.xhtml

CONTRIBUTORS

BF (PhD), IM (PhD), TR (E.Prof), DA (Prof) all contributed to the design of the study. IM (PhD), BF (PhD), JO (PhD) were involved in data collection, and GP (PhD), IM (PhD), BF (PhD) were involved in data analysis. GP (PhD) drafted the manuscript. All authors contributed to the interpretation of the analysis, and critically revised and approved the manuscript. The authors would also like to acknowledge Dr Daisy Payne for her contribution in the data collection of several interviews and involvement in the very early stages of data analysis. The authors would also like to acknowledge the contribution of the wider team from the Programme Management Group of the Safe Use of Medicines Theme of the Yorkshire and Humber PSTRC.

CONTRIBUTORSHIP STATEMENT

- Dr George Peat is a Senior Research Fellow in Patient Safety employed by the University of Bradford as part of the National Institute for Health Research (NIHR) Yorkshire and Humber Patient Safety Translational Research Centre.
- Dr Beth Fylan is a Senior Lecturer in Patient Safety at the University of Bradford
- Dr Iuri Marques was a Senior Research Fellow in Patient Safety employed by the University of Bradford as part of the National Institute for Health Research (NIHR) Yorkshire and Humber Patient Safety Translational Research Centre at the time of the study.
- DK Theo Raynor is an Emeritus Professor at the University of Leeds
- David P Alldred is a Professor of Medicines Use and Safety at the University of Leeds.
- Dr Janice Olaniyan is a Research Fellow in Patient Safety employed by the University of Bradford as part of the National Institute for Health Research (NIHR) Yorkshire and Humber Patient Safety Translational Research Centre.

COMPETING INTERESTS

None to declare.

FUNDING

This research was funded by the National Institute for Health Research (NIHR) Yorkshire and Humber Patient Safety Translational Research Centre (NIHR Yorkshire and Humber PSTRC). The views expressed in this article are those of the authors and not necessarily those of the NIHR or the Department of Health and Social Care.

DATA SHARING STATEMENT

All data relevant to the study are included in the article.

ETHICS APPROVAL STATEMENT

Health Research Authority (HRA) and NHS Research Ethics Committee approval was obtained (Ref. 18/YH/0140).

For peer review only - http://bmjopen.bmj.com/site/about/guidelines.xhtml

REFERENCES

1. Clegg A, Young J, Iliffe S, et al. Frail in elderly people. *Lancet* 2013;2:752-62.

2. Scott, I A, Hilmer S N, Reeve E, et al. Reducing inappropriate polypharmacy: the process of deprescribing. *JAMA Intern Med* 2015;175:827-34.

3. Smith H, Miller K, Barnett, N, et al. Person-centred care including deprescribing for older people. *Pharmacy (Basel) 2019*;7:101.

4. Reeve E, Shakib S, Hendrix I, et al. Review of deprescribing processes and development of an evidence-based, patient-centred deprescribing process. *Br J Clin Pharmacol* 2014;78:738-47.

5. Jansen J, Naganathan V, Carter S M, et al. Too much medicine in older people? Deprescribing through shared decision making. *BMJ* 2016;3:353.

6. Thompson W, Lundby C, Graabaek, T, et al. Tools for deprescribing in frail older persons and those with limited life expectancy: A systematic review. *J Am Geriatr Soc* 2019;67:172-80.

7. Doherty A J, Boland P, Reed J, et al. Barriers and facilitators of deprescribing in primary care: a systematic review. *BJGP Open* 2020;4:1-14.

8. Sawan M, Reeve E, Turner J, et al. A systems approach to identifying the challenges of implementing deprescribing in older adults across different health-care settings and countries: a narrative review. *Expert Rev. Clin. Pharmacol.* 2020;13:233-45.

9. Gillespie R J, Harrison L, Mullan J. Deprescribing medications for older adults in the primary care context: A mixed studies review. *Health Sci Rep.* 2018;1:e45.

10. Clyne B, Smith S M, Hughes C M, et al. Sustained effectiveness of a multifaceted intervention to reduce potentially inappropriate prescribing in older patients in primary care (OPTI-SCRIPT study). *Implement Sci* 2016;11:79.

11. Linsky A, Simon S R, Marcello T B, et al. Clinical provider perceptions of proactive medication discontinuation. *Am J Manag Care*. 2015;21:277-83.

12. Green A R, Lee P, Reeve E, et al. Clinicians' perspectives on barriers and enablers of optimal prescribing in patients with dementia and coexisting conditions. *J Am Board Fam Med.* 2019;32:383-91.

13. Wallis K A, Andrews A, Henderson M. Swimming against the tide: primary care physicians' views on deprescribing in everyday practice. *Ann Fam Med.* 2017;15:341-46.

14. Weir K, Nickel B, Naganathan V, et al. Decision-making preferences and deprescribing: perspectives of older adults and companions about their medicines. *J Gerontol Series B.* 2017

15. Reeve E, Wolff J L, Skehan M, et al. Assessment of attitudes towards deprescribing in older medicare beneficiaries in the United States. *JAMA Intern Med.* 2018;178:1673-80.

16. Rieckert A, Sommerauer C, Krumeich, A, et al. Reduction of inappropriate medication in older populations by electronic decision support (the PRIMA-eDS study): a qualitative study practical implementation in primary care. *BMC Fam Pract.* 2018;19:110.

17. Sinnott C, Hugh S M, Boyce, M B, et al. What to give the patient who has everything? A qualitative study of prescribing for multimorbidity in primary care. *Br J Gen Pract.* 2017;67:501-06.

For peer review only - http://bmjopen.bmj.com/site/about/guidelines.xhtml

18. Linsky A, Meterko M, Bokhour B G, et al. Deprescribing in the context of multiple providers: Understanding patient preferences. *Am J Manag Care.* 2019;25:192-98.

19. Luijks H D, Loeffen M J W, Lagro-Janssen A L, et al. GP's considerations in multimorbidity management. *Br J Gen Pract*. 2012;62:503-10.

20. NHS England, BMA. *Investment and evolution: a five-year framework for GP contract reform to implement the NHS Long Term Plan.* January 2019. Available from: https://www.england.nhs.uk/wp-content/uploads/2019/01/gp-contract-2019.pdf

21. Clegg A, Bates C, Young J, et al. Development and validation of an electronic frailty index using routine primary care electronic health record data. *Age and Ageing.* 2016;45:353-60.

22. Cane J, O'Connor D, Michie, S. Validation of the theoretical domains framework for use in behaviour change and implementation research. *Implementation Sci.* 2012;7:37.

23. Ritchie J, Spencer L. Qualitative data analysis for applied policy research. In: Bryman A, Burgess R G, eds. Analysing qualitative data. London: Routledge 1994: 173-94.

24. NHS England. *Structured medication reviews and medicines optimisation: guidance.* September 2020. Available from: https://www.england.nhs.uk/wp-content/uploads/2020/09/SMR-Spec-Guidance-2020-21-FINAL-.pdf

25. Reeve E, To J, Hendrix I, et al. Patient barriers to and enables of deprescribing: a systematic review. *Drugs Ageing.* 2013;30:793-807.

Figure One- Diagram mapping the themes and sub-themes identified related to facilitators of deprescribing

Figure Two: Diagram mapping the themes and sub-themes identified related to barriers of deprescribing

For peer review only

For peer review only - http://bmjopen.bmj.com/site/about/guidelines.xhtml

Figure 1: Facilitators of deprescribing

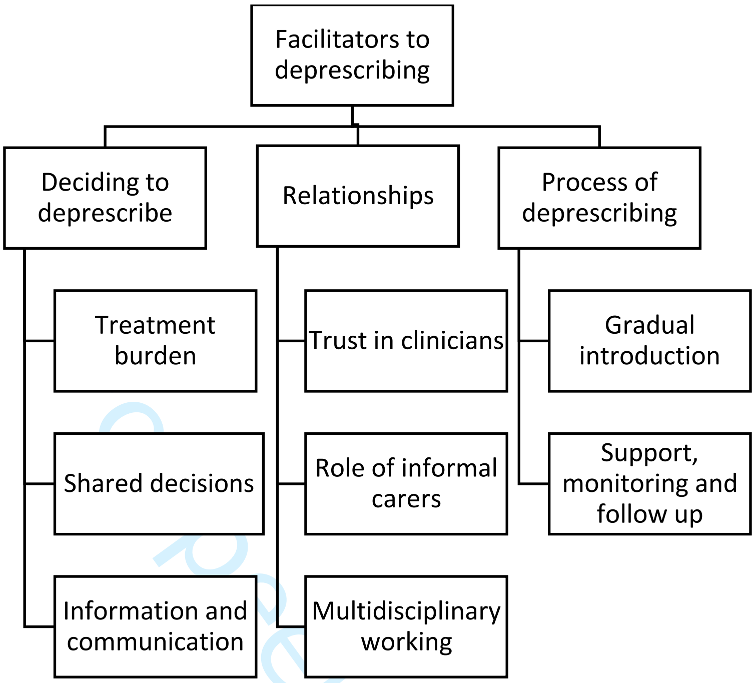

Figure 2- Barriers to deprescribing

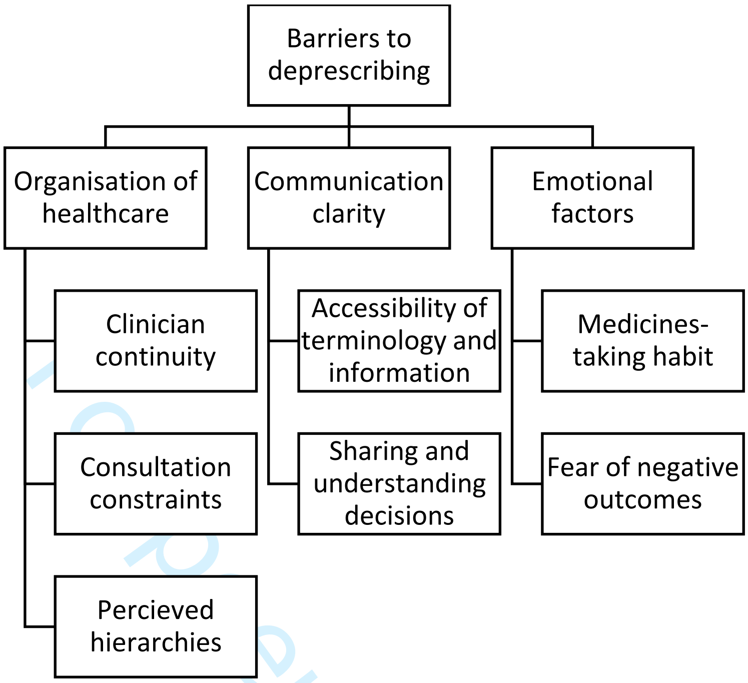

COREQ (COnsolidated criteria for REporting Qualitative research) Checklist

A checklist of items that should be included in reports of qualitative research. You must report the page number in your manuscript where you consider each of the items listed in this checklist. If you have not included this information, either revise your manuscript accordingly before submitting or note N/A.

Topic	Item No.	Guide Questions/Description	Reported on Page No.
**Domain 1: Research team and reflexivity**			
Personal characteristics			
Interviewer/facilitator	1	Which author/s conducted the interview or focus group?	19 (Contributor
Credentials	2	What were the researcher's credentials? E.g. PhD, MD	19 (Contributor
Occupation	3	What was their occupation at the time of the study?	19
Gender	4	Was the researcher male or female?	N/A
Experience and training	5	What experience or training did the researcher have?	4
Relationship with participants			
Relationship established	6	Was a relationship established prior to study commencement?	N/A
Participant knowledge of the interviewer	7	What did the participants know about the researcher? e.g. personal goals, reasons for doing the research	N/A
Interviewer characteristics	8	What characteristics were reported about the inter viewer/facilitator? e.g. Bias, assumptions, reasons and interests in the research topic	N/A
**Domain 2: Study design**			
Theoretical framework			
Methodological orientation and Theory	9	What methodological orientation was stated to underpin the study? e.g. grounded theory, discourse analysis, ethnography, phenomenology, content analysis	5
Participant selection			
Sampling	10	How were participants selected? e.g. purposive, convenience, consecutive, snowball	4
Method of approach	11	How were participants approached? e.g. face-to-face, telephone, mail, email	4
Sample size	12	How many participants were in the study?	5
Non-participation	13	How many people refused to participate or dropped out? Reasons?	N/A
Setting			
Setting of data collection	14	Where was the data collected? e.g. home, clinic, workplace	4
Presence of non-participants	15	Was anyone else present besides the participants and researchers?	5
Description of sample	16	What are the important characteristics of the sample? e.g. demographic data, date	6,7 (Table 1,2)
Data collection			
Interview guide	17	Were questions, prompts, guides provided by the authors? Was it pilot tested?	4
Repeat interviews	18	Were repeat inter views carried out? If yes, how many?	4
Audio/visual recording	19	Did the research use audio or visual recording to collect the data?	4
Field notes	20	Were field notes made during and/or after the inter view or focus group?	N/A
Duration	21	What was the duration of the inter views or focus group?	4
Data saturation	22	Was data saturation discussed?	N/A
Transcripts returned	23	Were transcripts returned to participants for comment and/or	N/A

For peer review only

Topic	Item No.	Guide Questions/Description	Reported on Page No.
		correction?	
**Domain 3: analysis and findings**			
Data analysis			
Number of data coders	24	How many data coders coded the data?	5
Description of the coding tree	25	Did authors provide a description of the coding tree?	Figure 1,2
Derivation of themes	26	Were themes identified in advance or derived from the data?	5
Software	27	What software, if applicable, was used to manage the data?	N/A
Participant checking	28	Did participants provide feedback on the findings?	N/A
Reporting			
Quotations presented	29	Were participant quotations presented to illustrate the themes/findings? Was each quotation identified? e.g. participant number	14-16
Data and findings consistent	30	Was there consistency between the data presented and the findings?	14-16
Clarity of major themes	31	Were major themes clearly presented in the findings?	14-16
Clarity of minor themes	32	Is there a description of diverse cases or discussion of minor themes?	14-16

Developed from: Tong A, Sainsbury P, Craig J. Consolidated criteria for reporting qualitative research (COREQ): a 32-item checklist for interviews and focus groups. *International Journal for Quality in Health Care*. 2007. Volume 19, Number 6: pp. 349 – 357

Once you have completed this checklist, please save a copy and upload it as part of your submission. DO NOT include this checklist as part of the main manuscript document. It must be uploaded as a separate file.

For peer review only

BMJ Open

Barriers and facilitators of successful deprescribing as described by older patients living with frailty, their informal carers, and clinicians. A qualitative interview study.

Journal:	*BMJ Open*
Manuscript ID	bmjopen-2021-054279.R1
Article Type:	Original research
Date Submitted by the Author:	13-Dec-2021
Complete List of Authors:	Peat, George; University of Bradford, School of Health and Life Sciences<br>Fylan, Beth; University of Bradford, School of Pharmacy and Medical Sciences; Bradford Institute for Health Research Yorkshire Quality and Safety Research Group<br>Marques, Iuri; University of Bradford, School of Pharmacy and Medical Sciences<br>Raynor, David; University of Leeds, School of Healthcare<br>Breen, Liz; University of Bradford, School of Pharmacy and Medical Sciences<br>Olaniyan, Janice; University of Bradford, School of Health and Life Sciences<br>Alldred, David; University of Leeds, School of Healthcare
**Primary Subject Heading**:	General practice / Family practice
Secondary Subject Heading:	General practice / Family practice, Qualitative research
Keywords:	PRIMARY CARE, QUALITATIVE RESEARCH, GERIATRIC MEDICINE

SCHOLARONE™
Manuscripts

I, the Submitting Author has the right to grant and does grant on behalf of all authors of the Work (as defined in the below author licence), an exclusive licence and/or a non-exclusive licence for contributions from authors who are: i) UK Crown employees; ii) where BMJ has agreed a CC-BY licence shall apply, and/or iii) in accordance with the terms applicable for US Federal Government officers or employees acting as part of their official duties; on a worldwide, perpetual, irrevocable, royalty-free basis to BMJ Publishing Group Ltd ("BMJ") its licensees and where the relevant Journal is co-owned by BMJ to the co-owners of the Journal, to publish the Work in this journal and any other BMJ products and to exploit all rights, as set out in our licence.

The Submitting Author accepts and understands that any supply made under these terms is made by BMJ to the Submitting Author unless you are acting as an employee on behalf of your employer or a postgraduate student of an affiliated institution which is paying any applicable article publishing charge ("APC") for Open Access articles. Where the Submitting Author wishes to make the Work available on an Open Access basis (and intends to pay the relevant APC), the terms of reuse of such Open Access shall be governed by a Creative Commons licence – details of these licences and which Creative Commons licence will apply to this Work are set out in our licence referred to above.

Other than as permitted in any relevant BMJ Author's Self Archiving Policies, I confirm this Work has not been accepted for publication elsewhere, is not being considered for publication elsewhere and does not duplicate material already published. I confirm all authors consent to publication of this Work and authorise the granting of this licence.

For peer review only

Barriers and facilitators of successful deprescribing as described by older patients living with frailty, their informal carers, and clinicians. A qualitative interview study.

Authors: George Peat [1,2,4], Beth Fylan [1,2,4,5], Iuri Marques[1,2,4], DK Theo Raynor [2,3], Liz Breen [1,2,4], Janice Olaniyan [1,2,4], David P Alldred [2,3,4].

*Corresponding author: George Peat ORCID 0000-0002-0293-2456. Email: gwpeat@bradford.ac.uk. Address: George Peat, School of Pharmacy and Medical Sciences, University of Bradford, Bradford, BD7 1DP, UK.

1. School of Pharmacy and Medical Sciences, University of Bradford, Bradford, UK.

2. NIHR Yorkshire and Humber Patient Safety Translational Research Centre (YH PSTRC), Bradford, UK.

3. School of Healthcare, University of Leeds, Leeds, UK.

4. Wolfson Centre for Applied Health Research, Bradford, UK

5. Yorkshire Quality and Safety Research Group, Bradford Institute for Health Research, Bradford, UK.

Key words: Primary care, Qualitative research, Geriatric medicine

Word count: 4196

For peer review only

For peer review only - http://bmjopen.bmj.com/site/about/guidelines.xhtml

TITLE: Barriers and facilitators of successful deprescribing as described by older patients living with frailty, their informal carers, and clinicians. A qualitative interview study.

AUTHORS:

Peat G, Fylan B, Marquis I, Raynor DK T, Breen L, Alldred D P, Olaniyan J.

ABSTRACT

Objective

To explore the barriers/facilitators to deprescribing in primary care in England from the perspectives of clinicians, patients living with frailty who reside at home, and their informal carers, drawing on the Theoretical Domains Framework (TDF) to identify behavioural components associated with barriers/facilitators of the process.

Design

Exploratory qualitative study

Setting

General practice (primary care) in England

Participants

9 patients aged 65+ living with frailty who attended a consultation to reduce or stop a medicine/s. 3 informal carers of patients living with frailty. 14 primary care clinicians including GPs, practice pharmacists, and advanced nurse practitioners.

Methods

Qualitative semi-structured interviews took place with patients living with frailty, their informal carers, and clinicians. Patients (n=9) and informal carers (n=3) were interviewed twice: immediately after deprescribing, and five/six weeks later. Clinicians (n=14) were interviewed once. In total, 38 interviews were undertaken. (). Framework analysis was applied to manage and analyse the data.

Results

Six themes associated with facilitators and barriers to deprescribing were generated respectively, with each supported by between two and three sub-themes. Identified facilitators of deprescribing with patients living with frailty included shared decision making, gradual introduction of the topic, clear communication of the topic to the patient, and multidisciplinary working. Identified barriers of deprescribing included consultation constraints, patients fear of negative consequences, and inaccessible terminology and information.

Conclusions

This paper offers timely insight into the barriers and facilitators to deprescribing for patients living with frailty within the context of primary care in England. As deprescribing continues to grow in national and international significance, it is important that future deprescribing interventions acknowledge the current barriers and facilitators and their associated behavioural components experienced by clinicians, patients living with frailty, and their informal carers to improve the safety and effectiveness of the process.

STRENGTHS AND LIMITATIONS OF THE STUDY

For peer review only - http://bmjopen.bmj.com/site/about/guidelines.xhtml

- A strength and novel aspect of the study was interviewing patients about their deprescribing journey at two timepoints, immediately after their deprescribing consultation and 4 to 6 weeks later.
- A strength of the study was the inclusion of patients, their informal carers, and healthcare professionals collectively, providing a multi-perspective insight into the barriers and facilitators of deprescribing in primary care in England.
- Our sample of patients and informal carers was lower than expected, due to challenges recruiting patients who were actively having a medicine deprescribed.

For peer review only

For peer review only - http://bmjopen.bmj.com/site/about/guidelines.xhtml

INTRODUCTION

Frailty can be understood as the age-related decline in physiological structures that can result in, among other consequences, an increased vulnerability to adverse drug reactions (ADRs). This is due to the interplay between physiological changes and pharmacokinetics and pharmacodynamics.(1) Consequently, there is an increasing focus on the safe and appropriate use of medicines in patients living with frailty. One strategy to improve medicines safety is to reduce or stop medicines that may no longer be appropriate or beneficial, a process referred to as deprescribing.(2)

If undertaken appropriately, deprescribing has been shown to support a reduction in ADRs and can improve quality of life.(3) Several process tools to support deprescribing have been created (e.g. 2,4-6). However, to date, no implementation science frameworks exist specific to deprescribing. Such a framework may allow researchers to implement deprescribing, with the knowledge of the contextual determinants in a specific setting, to cater the process to the specific needs of the practice population (7). Furthermore, identifying factors that facilitate or hinder deprescribing continues to be a high priority to ensure the process of deprescribing is safely and effectively implemented to optimise patient outcomes.(8)

Evidence suggests that barriers to deprescribing exist at various socio-ecological levels.(8) For instance, several studies have found clinicians in primary care face time restraints that hinder effective deprescribing, including limited time to review medicines, engage in consultations, and provide adequate follow-up.(9-11) Interpersonal barriers also exist and include ineffective communication with prescribers across healthcare settings, with some prescribers hesitant to deprescribe medicines prescribed by specialists in other care settings.(10, 12, 13)Challenges with communicating deprescribing effectively to patients using appropriate discourse exist.(12) Similarly, studies have reported prescriber hesitancy to deprescribe due to limited knowledge and guidance on how to do so appropriately and safely.(9; 14, 15) Finally, clinician hesitancy to deprescribe also exists because of a fear that deprescribing may be perceived by the patient as a withdrawal of care.(9)

From the perspective of patients living with frailty, managing multiple morbidities often means engaging with multiple prescribers over a sustained period, with evidence suggesting this scenario can make deprescribing challenging. For instance, an increased risk of poor communication between parties involved in a patient's care has been cited, resulting in patients confused about which prescriber has authority to deprescribe.(10) Similarly, patients prescribed medicines for a significant period are often reluctant to stop those medicines, particularly if they believe that the medicine is necessary, or that stopping the medicine would result in negative outcomes.(9;16) Likewise, older adults often lack sufficient knowledge about their medicines, limiting confidence and willingness to engage in decision making related to deprescribing.(15) Finally, paternalistic prescribing attitudes, void of shared decision-making, have also been shown to be a barrier to deprescribing.(15,16)

Studies have also reported facilitators of deprescribing. These include ensuring clinicians receive sufficient information and support related to the risks and benefits of deprescribing specific medicines to enable an informed decision.(14) Similarly, adopting an organisational culture, which encourages peer-to-peer learning, has been found to improve the self-efficacy of clinicians to deprescribe.(12,17) From a patient perspective, factors such as shared decision-making, trust in the clinician as a result of familiarity built through sustained clinician-patient relations, and a desire to reduce medicines have been found to support deprescribing.(10, 18-20)

For peer review only - http://bmjopen.bmj.com/site/about/guidelines.xhtml

Despite a growing evidence-base on barriers to and facilitators of deprescribing, studies largely exist outside of the UK with only three out of forty studies identified in a recent systematic review on deprescribing in primary care.(8) More so, there is a paucity in research into deprescribing focused specifically on patients living with frailty, a patient cohort increasingly considered a priority group for interventions that improve medicines safety. For example, recent policy in England, such as the General Medical Services (GMS) contract, requires General Practice (GP) to conduct Structured Medication Reviews (SMR) with patients living with moderate to severe frailty.(21) A potential outcome of an SMR is that a patient may have one or more of their medicines stopped. To support the success and effectiveness of deprescribing as a potential outcome of SMRs for patients living with frailty, clinicians need to be aware of the barriers to and facilitators of deprescribing in this group. Furthermore, while international studies have aimed to understand the patient perspective on deprescribing, no studies appear to have captured the barriers/facilitators of deprescribing from the perspective of patients who were actively having a medicine reduced or stopped. Finally, few studies have identified the determinants of behaviour that either facilitate, or act as a barrier to deprescribing. To address these gaps, this study aimed to explore the barriers/facilitators to deprescribing from the perspectives of clinicians, patients living with frailty who reside at home and were candidates for deprescribing, and their informal carers in the specific context of primary care in England, drawing on the Theoretical Domains Framework (TDF) to identify behavioural components associated with barriers/facilitators of the process.

METHODS

Study design, setting, and sampling (inclusion/exclusion)

The study adopted a qualitative design and recruited from four GP practices across regions of Yorkshire and Humber in England. Purposeful sampling was used to recruit patients ≥ 65 years old, living with frailty (either formally diagnosed and registered in the primary care electronic health record or at risk of frailty as defined by the electronic Frailty Index, eFI embedded within GP prescribing systems (22) who attended a consultation to deprescribe. In addition, informal carers who provided informal care to patient participants were also recruited to the study. Clinicians recruited eligible patients if during their appointment a medicine had been identified for deprescribing. Patients were provided an invitation letter and participant information sheet. Those who agreed to participate gave signed, informed consent. Patients were asked if they had an informal carer and invitations were extended to them.

To capture the perspective of clinicians, healthcare professionals involved in deprescribing (GPs, pharmacists, nurses and pharmacy technicians) were interviewed. Clinicians were recruited from the four participating surgeries. They were approached by a practice representative and were invited to take part, received an invitation letter and participant information sheet. Those who agreed to participate gave signed, informed consent. All clinicians had experience of deprescribing.

This qualitative study is part of a larger programme of work aimed at enhancing the process of deprescribing with patients living with frailty in the primary care setting in England, undertaken by the ‘Safe Use of Medicines’ research theme, part of the NIHR Yorkshire & Humber Patient Safety Translational Research Centre.

Data collection

Semi-structured interviews were conducted between September 2018 and February 2019. Participants provided consent for the interviews to be audio-recorded and transcribed verbatim. Interviews with clinicians guided by an interview schedule (supplementary file

For peer review only - http://bmjopen.bmj.com/site/about/guidelines.xhtml

'interview schedule clinicians') took place at their practice and explored their experiences of deprescribing. Four interview schedules (supplementary files 'interview schedule patients 1st int', 'interview schedule patients 2nd int', 'interview schedule informal carers first int'. 'interview schedule informal carers 2nd int'), informed by the literature and the Theoretical Domains Framework (TDF), and developed in consultation with a patient and public involvement and engagement member (PPIE), were designed to capture experiences of deprescribing and potential barriers and facilitators during the process from the perspective of patients and their informal carers.(23) The TDF, which is a theoretical framework used to understand behavioural influences, was used to focus and facilitate discussions about the determinants of behaviours of clinicians, patients, and their informal carers. Patients and their informal carers were interviewed twice at their homes, up to one week after their appointment, and five-six weeks later. The first interview explored patients' immediate perceptions of deprescribing, while the second focused on outcomes and their experience of the process. Interviews lasted on average 30 minutes. Interviews were conducted by experienced healthcare researchers from a range of backgrounds: a psychologist experienced in pharmacy practice and patient safety research, a sociologist, and a pharmacist. All interviewers used the same interview guide and regular discussions were held to compare experiences of the interviews and discuss data as it was collected.

Data analysis

Framework analysis was applied to analyse the data.(24) The approach provides a practical and structured approach to data management and analysis through five stages of Framework Analysis: data familiarisation; iterative development of a framework for the purpose of data management; indexing of individual transcript data to the categories within the framework; summarising the data within each category; interpretation of the data.(24)Data was coded by GP, BF, and IM, with LB, DA, DKR, and JO providing feedback on the coding framework. Two broad master themes representative of the barriers and facilitators to deprescribing experienced by participants were first identified. Each broad theme was then explored to identify themes and sub-themes representative of a barrier or facilitator of deprescribing. Finally, each sub-theme was mapped to the TDF to identify domains to target for deprescribing interventions. Data were managed using Microsoft Excel.

Patient and public involvement

All patient-facing recruitment materials, including the topic guide/s, were reviewed by a patient public involvement and engagement (PPIE) representative. The PPIE representative was an experienced local lay contributor and a lay leader within the National Institute for Health Research (NIHR) Yorkshire and Humber Patient Safety Translational Research Centre.

RESULTS

38 interviews in total were undertaken. Nine patients were interviewed at two separate points, up to one week after appointment and five to six weeks later, along with three informal carers (Table 1). Fourteen clinicians were also interviewed once, comprising of 6 GPs, 2 practice pharmacists, 5 practice nurses and advance nurse practitioners, and 1 pharmacy technician (Table 2). Analysis of the data identified three themes associated with facilitators of deprescribing, with each theme supported by between two and three sub-themes (Figure 1). Three themes associated with barriers of deprescribing were also identified and were similarly supported by between two and three sub-themes (Figure 2). Tables 3 and 4 illustrate the themes and sub-themes identified, with each mapped onto the TDF to identify behavioural components associated with barriers and facilitators of deprescribing. Themes and sub-themes associated with facilitators to deprescribing were attributed to the following TDF domains: *Skills, Intentions, Belief about capabilities,*

For peer review only - http://bmjopen.bmj.com/site/about/guidelines.xhtml

Knowledge, Social influences, Social/professional role and identity, Environmental context and resource, Memory, attention, and decision processes, Goals. Conversely, the themes and sub-themes associated with barriers to deprescribing were attributed to the following domains: *Social influences, Environmental context and resources, Social/professional role and identity, Memory, attention, and decision processes, Skills (interpersonal), Emotion.*

Each whole theme and their sub-themes representative of a barrier, or facilitator of successful deprescribing are summarised in the next section.

For peer review only

For peer review only - http://bmjopen.bmj.com/site/about/guidelines.xhtml

Table 1- Demographics: Patients and informal carers

#	Patient Code	Age	Gender	Ethnicity	Number of medicines before deprescribing	Informal carers	
						Code	Relationship
**1**	Practice 1- Patient1	Early 70s	Male	White British	5	N/a	
**2**	Practice 1- Patient 2	Late 80s	Male	White British	15	N/a	
**3**	Practice 1— Patient 3	Late 80s	Male	White British	8	N/a	
**4**	Practice 2- Patient 1	Early 90s	Male	White British	7	N/a	
**5**	Practice 2 - Patient 2	Mid 80s	Male	White British	5	N/a	
**6**	Practice 3 - Patient 2	Early 70s	Female	White British	15	N/a	
**7**	Practice 3 - Patient 3	Late 70s	Female	White British	9	Practice 3 -IC3	Daughter
**8**	Practice 4 - Patient 1	Late 70s	Female	White British	16	Practice 4-IC1	Daughter-in-law
**9**	Practice 4 - Patient 3	Late 70s	Male	White British	14	Practice 4-IC3	Wife

Table 2- Demographics: Clinicians

#	HCP Code	Gender	Ethnicity	Job Role	Prescriber	Years and Type of Experience
**1**	Practice 1-HCP1	Female	White British	GP	Yes	15 years as a doctor, 8 years as a GP
**2**	Practice 1-HCP2	Female	White British	Practice Pharmacist	No	34 years as a pharmacist, 9 months in primary care
**3**	Practice 1-HCP3	Female	White British	Practice Nurse	Yes	23 years as a nurse, 14 years in primary care, 15 months as prescriber
**4**	Practice 2-HCP1	Female	White British	GP	Yes	25 years as a doctor, 22 years as a GP
**5**	Practice 2-HCP2	Female	White British	Pharmacy Technician	No	32 Years as pharmacy technician, 6 years in primary care
**6**	Practice 2-HCP3	Female	White British	Advanced Nurse Practitioner	Yes	20 years as a nurse, 15 years in primary care, 3 years as prescriber
**7**	Practice 3-HCP1	Female	White British	Advanced Nurse Practitioner	No	44 years as a nurse, 10 years in primary care
**8**	Practice 3-HCP2	Female	White British	GP	Yes	28 years as a doctor, 23 years as a GP
**9**	Practice 3-HCP3	Male	White British	GP	Yes	11 years as a doctor, 6 years as GP
**10**	Practice 3-HCP4	Female	White British	GP	Yes	20 years as a doctor, 14 years as GP
**11**	Practice 4-HCP1	Female	White British	GP	Yes	7 years as a doctor, 1.5 years as a GP
**12**	Practice 4-GCP2	Female	White British	Practice Nurse	Yes	29 years as a nurse, 12 years as practice Nurse, 8 years as a prescriber
**13**	Practice 4-HCP3	Female	White British	Practice Nurse	Yes	4 years as a nurse, 2 years in primary care and as prescriber
**14**	Practice 4-HCP4	Female	White British	Practice Pharmacist	No	13 years as a pharmacist, 2.5 years in primary care

For peer review only - http://bmjopen.bmj.com/site/about/guidelines.xhtml

Table 3- Facilitators of deprescribing

Table 3. Facilitators of deprescribing			
**Theme**	**Sub-theme**	**TDF domain**	**Sample quotes**
**Deciding to deprescribe**	Treatment burden	Intentions	"I'm glad those things have reduced". P2 (Practice 1) "We get quite a few people who are on a lot of medication, don't generally know why they take certain ones. And you do get a lot of them saying is there any of these that I could actually stop?" HCP2 (Practice 4).
	Shared decisions	Belief about capabilities Skills (ability)	"I am a great believer in shared decision making so I do spend a lot of my consultations around making sure that [the patient knows the reason for taking the medicine], because their concordance (adherence) will be very poor if they don't understand why they are taking it and they need to understand the importance." HCP3 (Practice 1).
	Information and communication	Skills (interpersonal) Knowledge (procedural)	"…you're doing something that they ultimately disagree with and you're telling them that they can't have something that they want, that's the crux of the matter, but you also have to use your communication skills to explain why you're doing that, why is it that you're stopping this medication." HCP3 (Practice 3).
**Relationships**	Trust in clinicians	Skills (interpersonal)	) "So, I think for this lady I knew her, so I had a relationship with her which helps. And I think building that understanding and trust between us, so I think it was positive that we had trust…" HCP4 (Practice 4)
	Role of informal carers	Social Influences (social support)	"She can't always take in what's being said. If I had known that they were going to have her in to take her medication away I'd have gone with her, so that I could listen to the explanations that were being given to her to allay her worries." IC1 (Practice 4)

For peer review only - http://bmjopen.bmj.com/site/about/guidelines.xhtml

Table 3. Facilitators of deprescribing (cont)			
**Theme**	**Sub-theme**	**TDF domain**	**Sample quotes**
**Relationships (cont)**	Multidisciplinary working	Social/professional role and identity (organisational commitment)<br>Environmental context and resource (organisational culture)	"We've done a lot of deprescribing work here and it has been successful because we decided to involve the whole team. It would be no good if I sat here in a practice with six GPs and decided to improve my deprescribing because the other five GPs would be quietly sabotaging that without realising because we're not working together. If we all change our deprescribing patterns, then we have a hope of really making [a] change." HCP1 (Practice 1)
**Process of deprescribing**	Gradual introduction	Skills (interpersonal)<br>Memory, attention, and decision processes<br>Reinforcement<br>Beliefs about consequences<br>Goals<br>Skills (competence)	"It was about a control thing, they start off with one thing, one little step, and then they came back and said how they felt about that step; for this case it was pregabalin, and I talked about how to reduce it and if actually they got side effects or if things got worse it was then to speak and actually go further; just wait a little while and then make your next step, and giving them back some of the control, so they felt in charge rather than me, and that always seems to work much better." HCP2 (Practice 3)
**Process of deprescribing (cont)**	Support, monitoring, follow up	Goals (target setting)<br>Knowledge (procedural)	"On that occasion [I received] as much [support] as I'd wanted, previously I'd discussed it [discontinuing Aspirin] with [my GP] and with other practitioners at the health centre and been very, felt very supported." P1 (Practice 1)

For peer review only - http://bmjopen.bmj.com/site/about/guidelines.xhtml

Table 4: Barriers to deprescribing

Table 4- Barriers to deprescribing			
**Theme**	**Sub-theme**	**TDF domain**	**Sample quotes**
**Organisation of healthcare**	Clinician continuity	Social influences<br>Environmental context and resources	"You can't get in touch with your Doctor. It's no good going to another Doctor that doesn't understand that you're coming off your morphine and how it's affecting you." P2 (Practice 3)<br><br>"You never see the same doctor twice (...) unless you're under one person all the time you could go haywire (...) That's often the way when you see one doctor after another, your frequent doctor gets changed. They jump in too quick, if you're under the care of one person then that person can really monitor you." P1 (Practice 2)
	Consultation constraints	Environmental context and resources	"We're in tough times in primary care and pressure of demand is really, really high and so ad-hoc deprescribing is really difficult to do." HCP1 (Practice 1).<br><br>"I think it might have been easier if I hadn't done it [reviewed patient's medicines] over the phone, it might have actually been easier with that patient in front of me, but I've got time limitations." HCP2 (Practice 1)<br><br>"They haven't got time you see; you go into the surgery and you've only 10 minutes." IC3 (Practice 4)
	Perceived hierarchies	Social/professional role and identity	"I can't remember the exact drug, but I can tell you it was a cardiology medication, and the resistance really was they'd been told, you know this Cardiologist had told them they needed it and they needed to have it lifelong." HCP1(Practice 4)

For peer review only - http://bmjopen.bmj.com/site/about/guidelines.xhtml

**Table 4. Barriers to Deprescribing (cont.)**			
**Theme**	**Sub-theme**	**TDF domain**	**Sample quotes**
**Communication clarity**	Accessibility of terminology and information	Memory, attention, and decision processes	"I think [patients] get a bit confused by the word deprescribing. It can be very much a clinical term. we need to accept that patients aren't [always familiar with clinical terms] and so I would quite often call it something different." HCP2 (Practice 2)<br><br>"My first impression was that [deprescribing] was something that was done to one not something that you had an active say in. Yeah but it was a decision that a healthcare professional took and told the patient about it." P1 (Practice 1)
	Sharing and understanding decisions	Memory, attention, and decision processes<br>Skills (interpersonal)	"I don't want them to say right we'll start reducing this, I need to know why and what's going to happen with me." P2 (Practice 3)<br>"They don't explain what they're trying to say to you. It's as if oh we'll see you next week, go on we'll see you again later. It's…not explained what they're telling you…" P3 (Practice 4).
**Habits and fears**	Medicines-taking habit	Emotion	"Because she [patient] panics. And she's been on them such long-term and it becomes habit. And I think when you stop something that you've been doing for a long time you think 'oh', you panic straight away, you think 'oh, it's all going to go wrong, it's all going to go wrong." IC1 (Practice 4).
	Fear of negative consequences of deprescribing	Emotion	"Yes, I was afraid. Because the morphine helped me with the pain and I knew each time I was coming down I was getting pain, so I just took paracetamol and then it just kept coming down I think it was about every month she wanted me to have a go at coming down." P2 (Practice 3).

For peer review only - http://bmjopen.bmj.com/site/about/guidelines.xhtml

Insert Figure 1

Insert Figure 2

Facilitators to Deprescribing

Theme One: Deciding to Deprescribe

Arriving at a decision to deprescribe a medicine(s) was facilitated by several factors. First, some patients wished to reduce their medicines, a viewpoint reflected both in the accounts of patients and clinicians (Table 3). To reach a deprescribing decision, clinicians also advocated shared decision-making during consultation:

> *"I am a great believer in shared decision making so I do spend a lot of my consultations around making sure that [the patient knows the reason for taking the medicine], because their concordance (adherence) will be very poor if they don't understand why they are taking it…" HCP3 (Practice 1).*

Ensuring a patient understood why deprescribing was appropriate was essential in maintaining adherence with a new regimen, with shared decision-making integral to developing this understanding. Similarly, effectively communicating why deprescribing was appropriate was important, particularly with patients who were initially hesitant to deprescribe (Table 3):

Theme two: Relationships

Positive relationships between involved parties were important to facilitating deprescribing. Established trust, built through a knowingness between patient and clinician was highlighted as a facilitator by participants (Table 3). In addition, involving informal carers in deprescribing consultations was cited as beneficial to assist patients where necessary to understand why their medicine/s were deprescribed. (Table 3). Furthermore, the relational dynamic between clinicians and their practice was cited as significant. For instance, participants described the benefits of adopting a practice agenda to deprescribing:

"We've done a lot of deprescribing work here and it has been successful because we decided to involve the whole team. It would be no good if I sat here in a practice with six GPs and decided to improve my deprescribing because the other five GPs would be quietly sabotaging that without realising because we're not working together. If we all change our deprescribing patterns, then we have a hope of really making [a] change" HCP1 (Practice 1)

Care for older people living with frailty often involves multiple clinicians and healthcare workers. Working across teams to inform those involved in the patient's care of the decision to deprescribe was important to supporting the patient to accept their new medicines regimen:

Participant: "I've brought in the specialist, I'd already got the agreement of the GP involved in the patient's care, this was about course of action and it was highlighted to the healthcare assistant who she normally sees as well that it was you know the incorrect treatment for the patient." HCP3 (Practice 3)

Interviewer: "What was the outcome?"
Participant: "She has now accepted that it's not the right course of treatment for her and it can actually cause more difficulties" HCP3 (Practice 3)

For peer review only - http://bmjopen.bmj.com/site/about/guidelines.xhtml

Theme three: Process of deprescribing

Treating deprescribing as a process, as opposed to a one-off event, was a key facilitator relayed by participants. Patients described feeling more assured and confident of the process if it was conveyed as a gradual reduction in their medicine(s). Gradually reducing dosage of medicines was associated with having a sense of control by some participants, indeed, clinicians expressed the importance of allowing the patient 'to feel in charge' of the process:

> *"It was about a control thing, they start off with one thing, one little step, and then they came back and said how they felt about that step; for this case it was pregabalin, and I talked about how to reduce it and if actually they got side effects or if things got worse it was then to speak and actually go further; just wait a little while and then make your next step, and giving them back some of the control, so they felt in charge rather than me, and that always seems to work much better" HCP2 (Practice 3)*

Deprescribing was described as 'much better' when patients were enabled to feel in control, a feeling that manifested from ensuring the patient was informed, and that steps to reduce their medicines were made tentatively in a controlled fashion:

> *"I think as long as you kind of go you know 'Obviously if you ever do need it again of course we'll restart it again you know, but let's just do it really slowly and we'll do it together and I'll keep an eye on you and you'll come and see me and we'll review you' I think most people are actually quite happy to at least give it a go and try and come off medication" HCP1 (Practice 2)*

Barriers

Theme one: Organisation of healthcare

Aspects of the structure and organisation of healthcare were perceived as barriers to deprescribing. Participants described difficulty in seeing the same clinician, without clinician continuity, some participants felt that they were unable to be sufficiently monitored, and their deprescribing journey would not be understood:

> *"You never see the same doctor twice... unless you're under one person all the time you could go haywire [frustrated]... That's often the way when you see one doctor after another. They jump in too quick. If you're under the care of one person then that person can really monitor you." Patient 1 (Practice 2)*

Time limitations associated with the consultation were a further barrier. Clinicians described how time demands placed on primary care did not facilitate ad-hoc deprescribing. Similarly, time limitations influenced the type of deprescribing consultations clinicians offered. For example, one participant expressed that while face-to-face consultations were 'easier,' due to time limitations, reviews often took place over the telephone:

> *"I think it might have been easier if I hadn't done it [reviewed patient's medicines] over the phone, it might have actually been easier with that patient in front of me, but I've got time limitations" HCP2 (Practice 1)*

Time limitations on consultations were also voiced by informal carers, with the perception that clinicians 'haven't got the time':

For peer review only - http://bmjopen.bmj.com/site/about/guidelines.xhtml

“They haven’t got time you see; you go into the surgery and you’ve only 10 minutes" IC 3 (Practice 4)

The perceived hierarchy associated with clinicians was also voiced as a barrier to deprescribing. Participants spoke of this being a particular issue when patients had been prescribed medicines by a specialist in the past, who may have conveyed that the medicine(s) were a ‘lifelong’ regimen:

> *"I can’t remember the exact drug, but I can tell you it was a cardiology medication, and the resistance really was they’d been told, you know this Cardiologist had told them they needed it and they needed to have it lifelong." HCP1 (Practice 4)*

Theme two: Communication clarity

Challenges to communicating deprescribing effectively were identified as barriers to deprescribing. First, the term ‘deprescribing’ was thought to be a ‘clinical term’ and therefore created an impression that it ‘*was something that was done to one, not something you had an active say in.’ (P1, Practice 1).* Clinicians also felt patients may feel *‘a bit confused by the word deprescribing.’ (HCP2, Practice 2).* More so, terminology was a particular barrier in settings where the practice had *‘quite a number of different populations with lots of different languages’ (HCP3, Practice 2).*

Communication barriers also related to the communicative make-up of the deprescribing consultation. For example, some patients felt that clinicians did not spend the time explaining why the decision to deprescribe may have been appropriate:

> *“They don’t explain what they’re trying to say to you. It’s as if oh we’ll see you next week. It’s…not explained what they’re telling you…” P3 (Practice 4).*

The importance of ensuring communication clarity in relation to the topic of deprescribing, particularly in the case of older people living with frailty was voiced by one clinician:

> *"I think sometimes people, if they’re slightly older, can worry that they’re just sort of, being written off”.* HCP2 (Practice 4)

Theme three: Habits and fears

The habitual nature of a patient’s medicines regimen, and fears of possible negative consequences from stopping medicines were reported to be barriers to deprescribing. For example, clinicians described how *"Some people don’t like change, there’s a resistance to change for some patients” (HCP3, Practice 1).* Patients described feeling ‘afraid’ of the potential side effects of reducing or stopping medicines. Furthermore, the habitual connection to medicines also caused some patients to ‘panic’ when such medicines were tapered or stopped:

> *"Because she [patient] panics. And she’s been on them such long-term and it becomes habit. And I think when you stop something that you’ve been doing for a long time you think ‘oh’, you panic straight away, you think ‘oh, it’s all going to go wrong, it’s all going to go wrong" IC1 (Practice 4).*

For peer review only - http://bmjopen.bmj.com/site/about/guidelines.xhtml

DISCUSSION

Several key findings were identified that contribute to the existing evidence base on deprescribing for patients living with frailty. First, and in relation to the TDF domains *environmental context and resources* and *social/professional role and identity*, adopting deprescribing as a whole practice/team agenda whereby resource is directed towards deprescribing is likely to lead to better outcomes. More so, encouraging multidisciplinary working appears to be particularly important to successfully manage deprescribing for patients living with frailty, who often have multiple morbidities managed by multiple clinicians. Ensuring all clinicians involved in the patient's care were aware of the decision to deprescribe, was found to be important in supporting the patient to accept their new medicines regimen. *Environmental and resource factors* were also found to hinder deprescribing efforts. Patients voiced frustrations related to the inability to see the same clinician. Furthermore, while instances of successful deprescribing consultations and follow-up were reported, so too were instances of patients feeling that they did not have the time to discuss the decision to deprescribe, with some patients also feeling dissatisfied about the follow-up care offered.

A key facilitator of deprescribing identified was the attribution of control to the patient throughout the deprescribing process. In relation to the TDF, domains related to *skills (interpersonal), goals*, and *belief about consequences*, were identified. For example, interpersonal skills were necessary to convey an attribution of control to the patient. Likewise, where clinicians, patients, and informal carers were able to agree on specific and gradual goals, patients' beliefs about the consequences of deprescribing were well managed. Furthermore, while some patients living with frailty may need to be supported by informal carers throughout the process, our findings suggest that patients benefit from feeling informed, engaged, and in control of the process of deprescribing. Identifying the TDF domains associated with barriers or facilitators of deprescribing, will help focus future intervention development aimed at improving deprescribing outcomes in elderly patients living with frailty. Of the 14 TDF domains, '*environmental context and resources', 'memory, attention, and decision processes' and 'skills (interpersonal)'* were the most identified by this study. Consequently, future interventions may target these behaviour domains by considering implementing prompts and cues for both healthcare professionals and patients, address multi-disciplinary patient-centredness and the patient's role in the deprescribing process.

Several limitations are also acknowledged. First, the number of patients and informal carers interviewed was lower than expected. Recruitment was challenging in relation to timing recruitment to when patients were having a medicine deprescribed. Similarly, the study could have benefitted from the inclusion of more informal carers. In addition, patients and their informal carers were interviewed together to support these patients to participate in the study. However, this is a potential limitation of the study, as interviewing informal carers and patients together could have limited collecting the negative experiences of both due to the other being present. These limitations notwithstanding, the data provided valuable insight into the real-time barriers and facilitators experienced by clinicians, patients, and their informal carers. Furthermore, a major strength, and novel aspect of this qualitative study was the fact that we interviewed patients at two-time points whilst they were *actually* having a medicine deprescribed. Interviewing patients immediately after consultation and six weeks later, provided useful insight into their experience of follow-up, highlighting practices that facilitate or hinder deprescribing. However, it is also acknowledged that only interviewing patients who were actively having a medicine deprescribed, as opposed to interviewing

For peer review only - http://bmjopen.bmj.com/site/about/guidelines.xhtml

patients living with frailty about deprescribing in general, may mean further barriers to deprescribing were not reported.

NHS England's implementation guidance for Structured Medication Reviews (SMRs) states the importance of shared decision-making and emphasises the SMR as an ongoing process as opposed to a one-off exercise.(25) Our findings support previous international evidence that highlights that patients over the age of 65 living with frailty want to be involved in decisions related to deprescribing.(19) Patients living with frailty are a priority cohort for SMRs, consequently, it is important that implementation guidance is acted on, particularly regarding ensuring patients are engaged in any deprescribing decisions made. Furthermore, while it is the case that patients may be unwilling to deprescribe if they believe their medicine is appropriate,(16) this study found some patients living with frailty were keen to engage in discussions about their medicines and how to reduce them, a finding shared elsewhere in the literature.(19) Consequently, these findings indicate that patients may be generally more open to the prospect of deprescribing than perceived.

Acknowledging the barriers to deprescribing as experienced by clinicians, patients living with frailty, and their informal carers is integral to improving the effectiveness and acceptability of the process. The term 'deprescribing' was perceived by patients as either confusing, or suggestive of a process led by the clinician as opposed to being shared. Similar concerns with deprescribing discourse have also been voiced in the literature(19) suggestive of a requirement to rethink how the process is conveyed to patients and the public. To successfully deprescribe, the views and concerns of patients need to be discussed as part of the consultation.(6,16, 26) Several participants voiced concerns about not understanding why their medicines were being stopped, a finding underreported in the current evidence base. Supporting patients to understand why their medicines are being deprescribed, may also reduce fears and concerns about the consequences of stopping/reducing medicines, a barrier found in this study and reported elsewhere in the literature (6,9). The shared commonalities in reported barriers/facilitators of deprescribing reported by this study and the wider literature highlight components of the process that future deprescribing intervention studies should seek to address, and those that should be prioritised to improve deprescribing effectiveness. Furthermore, identifying the barriers and facilitators of deprescribing and their associated behavioural domains, can support clinicians to deprescribe safely and effectively as part of the NHS mandated Structured Medicine Review programme.

In conclusion, this paper offers a timely contribution to the existing evidence base, providing insight into the barriers and facilitators to deprescribing experienced by clinicians, patients living with frailty and their informal carers within the context of primary care in England. As deprescribing continues to grow in national and international significance, it is important that future deprescribing interventions acknowledge the current barriers and facilitators and their associated behavioural components faced by clinicians, patients living with frailty, and their informal carers to improve the safety and effectiveness of the process.

For peer review only

CONTRIBUTORS

BF (PhD), IM (PhD), TR (E.Prof), LB (PhD) DA (Prof) all contributed to the design of the study. IM (PhD), BF (PhD), JO (PhD) were involved in data collection, and GP (PhD), IM (PhD), BF (PhD) were involved in data analysis. GP (PhD) drafted the manuscript. All authors contributed to the interpretation of the analysis, and critically revised and approved the manuscript.

CONTRIBUTORSHIP STATEMENT

- Dr George Peat is a Senior Research Fellow in Patient Safety employed by the University of Bradford as part of the National Institute for Health Research (NIHR) Yorkshire and Humber Patient Safety Translational Research Centre.
- Dr Beth Fylan is a Senior Lecturer in Patient Safety at the University of Bradford
- Dr Iuri Marques was a Senior Research Fellow in Patient Safety employed by the University of Bradford as part of the National Institute for Health Research (NIHR) Yorkshire and Humber Patient Safety Translational Research Centre at the time of the study.
- DK Theo Raynor is an Emeritus Professor at the University of Leeds
- Dr Liz Breen is a Reader in Health Service Operations at the University of Bradford
- David P Alldred is a Professor of Medicines Use and Safety at the University of Leeds.
- Dr Janice Olaniyan is a Research Fellow in Patient Safety employed by the University of Bradford as part of the National Institute for Health Research (NIHR) Yorkshire and Humber Patient Safety Translational Research Centre.

ACKNOWLEDGEMENTS

The authors would like to acknowledge Dr Daisy Payne for her contribution in the data collection of several interviews and involvement in the very early stages of data analysis. The authors would also like to acknowledge the contribution of the wider team from the Programme Management Group of the Safe Use of Medicines Theme of the Yorkshire and Humber PSTRC.

COMPETING INTERESTS

None to declare.

FUNDING

This research was funded by the National Institute for Health Research (NIHR) Yorkshire and Humber Patient Safety Translational Research Centre (NIHR Yorkshire and Humber PSTRC). The views expressed in this article are those of the authors and not necessarily those of the NIHR or the Department of Health and Social Care.

DATA SHARING STATEMENT

All data relevant to the study are included in the article.

ETHICAL APPROVAL STATEMENT

Health Research Authority (HRA) and NHS Research Ethics Committee approval was obtained (Ref. 18/YH/0140).

For peer review only - http://bmjopen.bmj.com/site/about/guidelines.xhtml

REFERENCES

1. Clegg A, Young J, Iliffe S, et al. Frail in elderly people. *Lancet* 2013;2:752-62.

2. Scott, I A, Hilmer S N, Reeve E, et al. Reducing inappropriate polypharmacy: the process of deprescribing. *JAMA Intern Med* 2015;175:827-34.

3. Smith H, Miller K, Barnett, N, et al. Person-centred care including deprescribing for older people. *Pharmacy (Basel) 2019*;7:101.

4. Reeve E, Shakib S, Hendrix I, et al. Review of deprescribing processes and development of an evidence-based, patient-centred deprescribing process. *Br J Clin Pharmacol* 2014;78:738-47.

5. Jansen J, Naganathan V, Carter S M, et al. Too much medicine in older people? Deprescribing through shared decision making. *BMJ* 2016;3:353.

6. Thompson W, Lundby C, Graabaek, T, et al. Tools for deprescribing in frail older persons and those with limited life expectancy: A systematic review. *J Am Geriatr Soc* 2019;67:172-80.

7. Ailabouni NJ, Reeve E, Helfrich CD, et al. Leveraging implementation science to increase the translation of deprescribing evidence into practice. *Res Social Adm Pharm.* 2021.doi: 10.1016/j.sapharm.2021.05.018

8. Doherty A J, Boland P, Reed J, et al. Barriers and facilitators of deprescribing in primary care: a systematic review. *BJGP Open* 2020;4:1-14.

9. Sawan M, Reeve E, Turner J, et al. A systems approach to identifying the challenges of implementing deprescribing in older adults across different health-care settings and countries: a narrative review. *Expert Rev. Clin. Pharmacol.* 2020;13:233-45.

10. Gillespie R J, Harrison L, Mullan J. Deprescribing medications for older adults in the primary care context: A mixed studies review. *Health Sci Rep.* 2018;1:e45.

11. Clyne B, Smith S M, Hughes C M, et al. Sustained effectiveness of a multifaceted intervention to reduce potentially inappropriate prescribing in older patients in primary care (OPTI-SCRIPT study). *Implement Sci* 2016;11:79.

12. Linsky A, Simon S R, Marcello T B, et al. Clinical provider perceptions of proactive medication discontinuation. *Am J Manag Care*. 2015;21:277-83.

13. Green A R, Lee P, Reeve E, et al. Clinicians' perspectives on barriers and enablers of optimal prescribing in patients with dementia and coexisting conditions. *J Am Board Fam Med.* 2019;32:383-91.

14. Wallis K A, Andrews A, Henderson M. Swimming against the tide: primary care physicians' views on deprescribing in everyday practice. *Ann Fam Med.* 2017;15:341-46.

15. Weir K, Nickel B, Naganathan V, et al. Decision-making preferences and deprescribing: perspectives of older adults and companions about their medicines. *J Gerontol Series B.* 2017

16. Reeve E, Wolff J L, Skehan M, et al. Assessment of attitudes towards deprescribing in older medicare beneficiaries in the United States. *JAMA Intern Med.* 2018;178:1673-80.

For peer review only - http://bmjopen.bmj.com/site/about/guidelines.xhtml

17. Rieckert A, Sommerauer C, Krumeich, A, et al. Reduction of inappropriate medication in older populations by electronic decision support (the PRIMA-eDS study): a qualitative study practical implementation in primary care. *BMC Fam Pract.* 2018;19:110.

18. Sinnott C, Hugh S M, Boyce, M B, et al. What to give the patient who has everything? A qualitative study of prescribing for multimorbidity in primary care. *Br J Gen Pract.* 2017;67:501-06.

19. Linsky A, Meterko M, Bokhour B G, et al. Deprescribing in the context of multiple providers: Understanding patient preferences. *Am J Manag Care.* 2019;25:192-98.

20. Luijks H D, Loeffen M J W, Lagro-Janssen A L, et al. GP's considerations in multimorbidity management. *Br J Gen Pract*. 2012;62:503-10.

21. NHS England, BMA. *Investment and evolution: a five-year framework for GP contract reform to implement the NHS Long Term Plan.* January 2019. Available from: https://www.england.nhs.uk/wp-content/uploads/2019/01/gp-contract-2019.pdf

22. Clegg A, Bates C, Young J, et al. Development and validation of an electronic frailty index using routine primary care electronic health record data. *Age and Ageing.* 2016;45:353-60.

23. Cane J, O'Connor D, Michie, S. Validation of the theoretical domains framework for use in behaviour change and implementation research. *Implementation Sci.* 2012;7:37.

24. Ritchie J, Spencer L. Qualitative data analysis for applied policy research. In: Bryman A, Burgess R G, eds. Analysing qualitative data. London: Routledge 1994: 173-94.

25. NHS England. *Structured medication reviews and medicines optimisation: guidance.* September 2020. Available from: https://www.england.nhs.uk/wp-content/uploads/2020/09/SMR-Spec-Guidance-2020-21-FINAL-.pdf

26. Reeve E, To J, Hendrix I, et al. Patient barriers to and enables of deprescribing: a systematic review. *Drugs Ageing.* 2013;30:793-807.

Figure One- Diagram mapping the themes and sub-themes identified related to facilitators of deprescribing

Figure Two: Diagram mapping the themes and sub-themes identified related to barriers of deprescribing

For peer review only

Figure 1: Facilitators of deprescribing

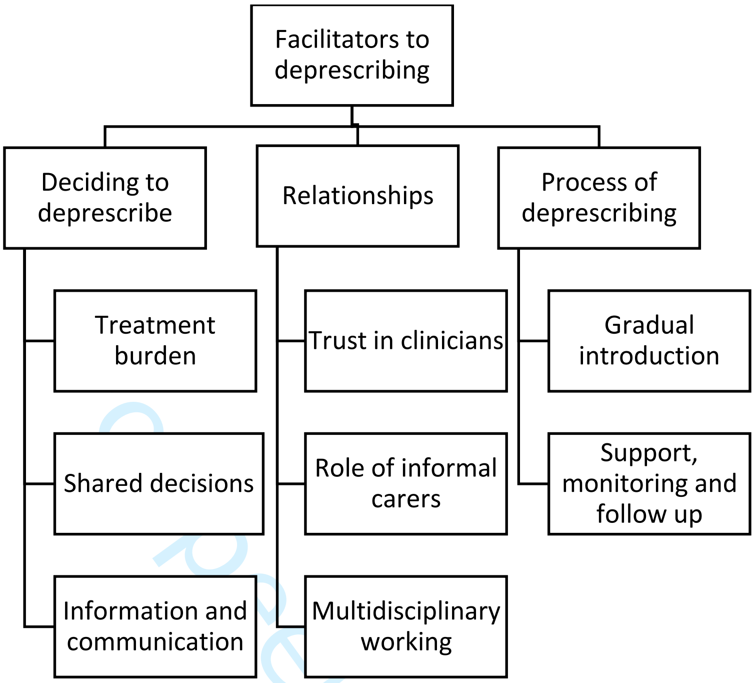

Figure 2- Barriers to deprescribing

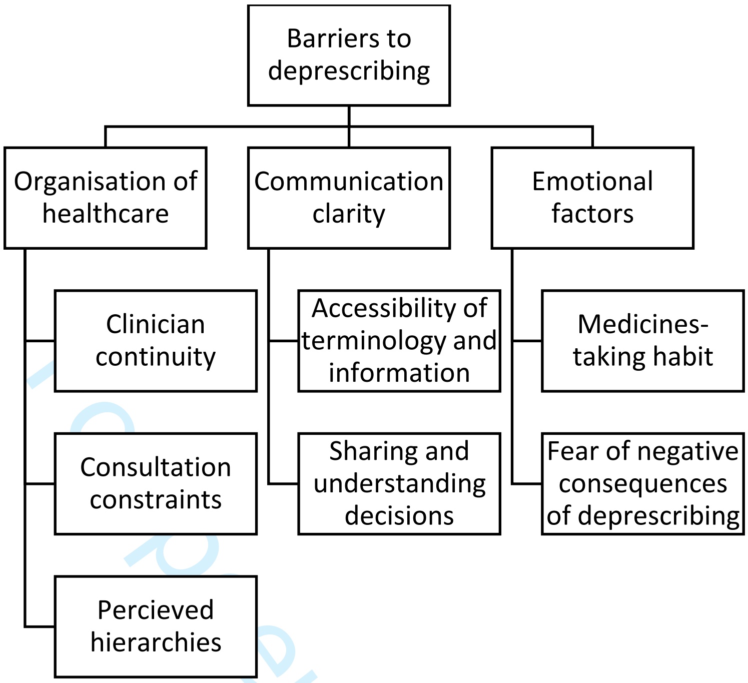

For peer review only

Qualitative interview: Patients (First interview): v.1.4

Qualitative interview: Patients: Interview 1	
**Topic**	**Questions**
**Deprescribing: Process**	I know you had one/a number of your regular medicines stopped by your HCP recently. Can you please tell me about this? Why did it happen, how and which medicine(s)? Whose decision was it to begin with? [If healthcare professional] Did you agree with the decision at the beginning?
	Did you feel you received enough information and support by your healthcare professional to make you feel comfortable about stopping these medicines? What went well and what could have gone better (during consultation)?
	Did you feel like you had a say in this decision? [Alternative] Were you given the opportunity to fully discuss your thoughts and explore your options?
	Do you think stopping a medicine is an easy decision to make? [If not] What makes it difficult to stop a medicine from your perspective? What do you think makes it difficult for your HCP? Are there any aspects which may facilitate this process? What do you think could be done to help stopping problematic medicines effectively?
**Deprescribing: Terminology**	Have you ever heard of the term deprescribing? Do you agree with the term? What comes to mind when you hear the word deprescribing? [Probe] what are your concerns? Would you rather call it something else? [If so] why? What makes you feel that way?
	What would you call the process of stopping medicines?
**Attitudes, beliefs, expectations about medicines and polypharmacy**	What are the challenges you experience with the daily management of your medicines? How well do you think you understand the medicines they you to take? What strategies do you use to ensure you take your medicines correctly?
**Patient-prescriber communication and relationship and decision-making**	Do you feel that you are involved in discussions and decisions about your medicines or do you usually defer decisions to your healthcare professional(s)? [if deferral] Why do you think this happens? And are you happy that decisions are deferred to your HCP?
	When making decisions about your medicines, do you often talk to your HCP about your personal care goals and priorities, particularly if you disagree with a decision? Does your HCP ask what your views are on the subject before making a decision? Have you ever disagreed with a decision made by your HCP about your medicines? [If so] Can you talk about it and what the outcome was?
	Do you feel supported and listened to by your healthcare professional(s) when you have questions or concerns about your medicines? [If not] Why not? Can you give me a recent example that made you feel this way?
**Final thoughts and considerations**	**Is there any other information, thought or concern you would like to share with me before we end this interview?**
**THANK YOU FOR TAKING PART IN THIS STUDY**	

For peer review only - http://bmjopen.bmj.com/site/about/guidelines.xhtml

Qualitative interview: Patients (Second interview): v.1.1

Interview Schedule – Patients 2nd interview	
**Topic**	**Questions**
**Deprescribing: Process**	In our last interview we discussed the appointment you had with your healthcare professional where one (or more) medicine(s) was (were) stopped. In these six (to eight) weeks since your appointment, did you experience any issues as a result of stopping this (these) medicine(s)? Can you talk a little bit about your experiences? What strategies did you use to help you overcome these difficulties? What were your concerns?
**Follow-up and on-going monitoring**	Did you seek professional (formal) help at any point? (e.g. GP, practice pharmacist or community pharmacist) If so, can you tell me how it happened? What steps were taken by your healthcare professional to provide you with support? What was the outcome (has it been resolved? How?)
	If (or when) you needed to contact a healthcare professional to discuss your condition and medicines in relation to the medicine(s) that was (were) stopped, were you provided with any contacts you could use? Alternatively: Did you know who to contact if you needed help?
	Were you contacted by your healthcare professional(s) at any point after your medicine(s) was (were) stopped)? If so, who contacted you? Why? How many times? If not, how important would it have been for your healthcare professional to contact you?
	Did you feel you received enough support after your medicine(s) was (were) stopped, or if you did not feel you needed it, do you feel you would have received adequate support if you needed? Who would you contact in the first instance if you needed to ask questions or raise concerns? Why? What was missing?
**Attitudes and behaviours towards deprescribing**	Six (to eight) weeks after you had your medicine(s) stopped, how do you feel? Probes: Would you consider stopping more medicines if your healthcare professional assured this would be good for your health or quality of life? What changed in your opinion comparing to when you stopped this (these) medicine(s) and if you were to stop another medicine in the future?
	What do you think was, or would have been crucial for succeeding in stopping your medicine (what went well and what could have gone better)? What happened that ensured the success? Is there anything else that might need to happen but did not which may have increased the chances of success? Is there anything you would have liked to happen between you and your healthcare professional during this time?
**Final thoughts and considerations**	Is there any other information, thought or concern you would like to share with me before we end this interview?
**THANK YOU FOR TAKING PART IN THIS STUDY**	

For peer review only - http://bmjopen.bmj.com/site/about/guidelines.xhtml

Qualitative interview: Informal carers (First interview): v.1.1

Qualitative interview: Informal carers. Interview 1	
**Topic**	**Questions**
**Deprescribing: Process**	I know the person you help with their medicines had one/a number of your regular medicines stopped by their HCP recently. Can you please tell me about this? Whose decision was it to begin with? [If healthcare professional] Did you agree with the decision? What are your concerns (if any)? Do you think your concerns are the same or different than those of your relative/friend?
	Did you feel you and your relative/friend received enough information and support from the healthcare professional to make you feel comfortable about stopping these medicines? What went well and what could have gone better (during consultation)?
	Were you given the opportunity to fully discuss your thoughts and explore your options?
	Do you think stopping a medicine is an easy decision to make? [If not] What makes it difficult to stop a medicine from your perspective? What do you think makes it difficult for your HCP? What do you think could be done to help stopping problematic medicines effectively?
**Deprescribing: Terminology**	Have you ever heard of the term deprescribing? Do you agree with the term? What comes to mind when you hear the word deprescribing? [Probe] what are your concerns? Would you rather call it something else? [If so] why? What makes you feel that way?
	What would you call the process of stopping medicines?
**Attitudes, beliefs, expectations about medicines and polypharmacy**	What are the challenges you and your relative/friend experience with his/her medicines? How well do you think you think your friend/relative understands the medicines he/she takes? What strategies do you use to ensure your friend/relative takes his/her medicines correctly?
**Patient-prescriber communication and relationship and decision-making**	Do you feel that you are involved in discussions and decisions about your friend/relative's medicines or do you usually defer decisions to the healthcare professional(s)? [if deferral] Why do you think this happens? And are you happy that decisions are deferred to the HCP?
	When making decisions about your relative/friend's medicines, do you often talk to your HCP about yours and your relative/friend's personal care goals and priorities, particularly if you disagree with a decision? Does the HCP ask what your views are on the subject before making a decision? Have you ever disagreed with a decision made by your HCP about your medicines? [If so] Can you talk about it and what the outcome was?
	Do you feel supported and listened to by your healthcare professional(s) when you have questions or concerns about your medicines? [If not] Why not? Can you give me a recent example that made you feel this way?
	Do you think your care goals and priorities for your relative/friend are the same as those of his/hers? If not, how do they differ?
**Final thoughts and considerations**	**Is there any other information, thought or concern you would like to share with me before we end this interview?**
**THANK YOU FOR TAKING PART IN THIS STUDY**	

For peer review only - http://bmjopen.bmj.com/site/about/guidelines.xhtml

Qualitative interview: Informal carers (Second interview): v.1.1

Qualitative interview: Informal carers: Interview 2	
**Topic**	**Questions**
**Deprescribing: Process**	In our last interview we discussed the appointment your friend/relative had with the healthcare professional where one (or more) medicine(s) was (were) stopped. In these six (to eight) weeks since this appointment, did your friend/relative experience any issues as a result of stopping this (these) medicine(s)? Can you talk a little bit about your experiences? What strategies did you use to help your relative/friend overcome these difficulties? What were your concerns?
**Follow-up and on-going monitoring**	Did you seek professional (formal) help at any point? (e.g. GP, practice pharmacist or community pharmacist) If so, can you tell me how it happened? What steps were taken by the healthcare professional to provide you with support? What was the outcome (has it been resolved? How?)
	If (or when) you needed to contact a healthcare professional to discuss your condition and medicines in relation to the medicine(s) that was (were) stopped, were you provided with any contacts you could use? Alternatively: Did you know who to contact if you needed help?
	Were you contacted by the healthcare professional(s) at any point after your relative/friend's medicine(s) was (were) stopped)? If so, who contacted you? Why? How many times? If not, how important would it have been for your healthcare professional to contact you?
	Did you feel you and your relative/friend received enough support after the medicine(s) was (were) stopped, or if you did not feel you needed it, do you feel you would have received adequate support if you needed? Who would you contact in the first instance if you needed to ask questions or raise concerns? Why? What was missing?
**Attitudes and behaviours towards deprescribing**	Six (to eight) weeks after your relative/friend had the medicine(s) stopped, how do you feel? Probes: What would you have done differently? What changed in your opinion comparing to when you stopped this (these) medicine(s)?
	What do you think was, or would have been crucial for succeeding in stopping medicines (what went well and what could have gone better)? What happened that ensured the success? Is there anything else that might need to happen but did not which may have increased the chances of success? Is there anything you would have liked to happen between you, your relative/friend and his/her healthcare professional during this time?
**Final thoughts and considerations**	Is there any other information, thought or concern you would like to share with me before we end this interview?
**THANK YOU FOR TAKING PART IN THIS STUDY**	

For peer review only - http://bmjopen.bmj.com/site/about/guidelines.xhtml

Qualitative interview: Clinicians: v.1.3

**Qualitative interview: Clinicians**	
**Topic**	**Questions**
**Deprescribing: Process**	Can you please talk about the last time you stopped a patients' medicine (or recommended it)? Probes: When did it happen, why, how and which medicine(s)? Who initiated the discussion?
	What information and support did you provide this patient to make him/her feel comfortable about stopping these medicines? What went well and what could have gone better?
	What thoughts and/or concerns did the patient discuss during the discussion to stop this/these medicine(s)? What options were there for the patient and how was the decision made?
	Did this patient experience any issues as a result of stopping this (these) medicine(s)? What strategies did you use to help him/her overcome these difficulties?
	Do patients ever ask you to have one or more medicines stopped? Can you tell me of an example of when this happened and how they initiated this discussion? What was the outcome?
	Do you think stopping a medicine is an easy decision to make? [If not] What makes it difficult to stop a medicine? What do you think makes it difficult for you? Are there any aspects which may facilitate this process?
**Deprescribing: Terminology**	How do you feel about the word deprescribing? Could you suggest an alternative name for it?
**Attitudes, beliefs, expectations about medicines and polypharmacy**	In your view, what are the challenges patients may experience with the daily management of their medicines? How well do you think older patients understand the medicines they have to take?
**Patient-prescriber communication and relationship and decision-making**	How do you ensure patients receive the necessary information about their medicines? Can you give me a recent example of when you ensured this?
	How often do you engage in discussions about your medicines with your patients? Who does normally initiate these discussions? Can you give me a recent example where a patient initiated a discussion about their medicines with you? [Probe] What was the outcome?
	When making decisions about patients' medicines, how do you ensure that patients' priorities are taken into consideration? Can you give me an example of when this happened? How often do patients agree or disagree with a decision you make about their medicines? [If so] Can you talk about it and what the outcome was?
**Final thoughts and considerations**	**Is there any other information, thought or concern you would like to share with me before we end this interview?**
	You have access to systems that help you make decisions about medicines e.g. STOP/START tool, Anticholinergic tool etc. How **do you feel** about using these tools if you do, and what are the **challenges** and **positives** of using them?
**THANK YOU FOR TAKING PART IN THIS STUDY**	

For peer review only - http://bmjopen.bmj.com/site/about/guidelines.xhtml

COREQ (COnsolidated criteria for REporting Qualitative research) Checklist

A checklist of items that should be included in reports of qualitative research. You must report the page number in your manuscript where you consider each of the items listed in this checklist. If you have not included this information, either revise your manuscript accordingly before submitting or note N/A.

Topic	Item No.	Guide Questions/Description	Reported on Page No.
**Domain 1: Research team and reflexivity**			
Personal characteristics			
Interviewer/facilitator	1	Which author/s conducted the interview or focus group?	19 (Contributor
Credentials	2	What were the researcher's credentials? E.g. PhD, MD	19 (Contributor
Occupation	3	What was their occupation at the time of the study?	19
Gender	4	Was the researcher male or female?	N/A
Experience and training	5	What experience or training did the researcher have?	4
Relationship with participants			
Relationship established	6	Was a relationship established prior to study commencement?	N/A
Participant knowledge of the interviewer	7	What did the participants know about the researcher? e.g. personal goals, reasons for doing the research	N/A
Interviewer characteristics	8	What characteristics were reported about the inter viewer/facilitator? e.g. Bias, assumptions, reasons and interests in the research topic	N/A
**Domain 2: Study design**			
Theoretical framework			
Methodological orientation and Theory	9	What methodological orientation was stated to underpin the study? e.g. grounded theory, discourse analysis, ethnography, phenomenology, content analysis	5
Participant selection			
Sampling	10	How were participants selected? e.g. purposive, convenience, consecutive, snowball	4
Method of approach	11	How were participants approached? e.g. face-to-face, telephone, mail, email	4
Sample size	12	How many participants were in the study?	5
Non-participation	13	How many people refused to participate or dropped out? Reasons?	N/A
Setting			
Setting of data collection	14	Where was the data collected? e.g. home, clinic, workplace	4
Presence of non-participants	15	Was anyone else present besides the participants and researchers?	5
Description of sample	16	What are the important characteristics of the sample? e.g. demographic data, date	6,7 (Table 1,2)
Data collection			
Interview guide	17	Were questions, prompts, guides provided by the authors? Was it pilot tested?	4
Repeat interviews	18	Were repeat inter views carried out? If yes, how many?	4
Audio/visual recording	19	Did the research use audio or visual recording to collect the data?	4
Field notes	20	Were field notes made during and/or after the inter view or focus group?	N/A
Duration	21	What was the duration of the inter views or focus group?	4
Data saturation	22	Was data saturation discussed?	N/A
Transcripts returned	23	Were transcripts returned to participants for comment and/or	N/A

For peer review only

Topic	Item No.	Guide Questions/Description	Reported on Page No.
		correction?	
**Domain 3: analysis and findings**			
Data analysis			
Number of data coders	24	How many data coders coded the data?	5
Description of the coding tree	25	Did authors provide a description of the coding tree?	Figure 1,2
Derivation of themes	26	Were themes identified in advance or derived from the data?	5
Software	27	What software, if applicable, was used to manage the data?	N/A
Participant checking	28	Did participants provide feedback on the findings?	N/A
Reporting			
Quotations presented	29	Were participant quotations presented to illustrate the themes/findings? Was each quotation identified? e.g. participant number	14-16
Data and findings consistent	30	Was there consistency between the data presented and the findings?	14-16
Clarity of major themes	31	Were major themes clearly presented in the findings?	14-16
Clarity of minor themes	32	Is there a description of diverse cases or discussion of minor themes?	14-16

Developed from: Tong A, Sainsbury P, Craig J. Consolidated criteria for reporting qualitative research (COREQ): a 32-item checklist for interviews and focus groups. *International Journal for Quality in Health Care*. 2007. Volume 19, Number 6: pp. 349 – 357

Once you have completed this checklist, please save a copy and upload it as part of your submission. DO NOT include this checklist as part of the main manuscript document. It must be uploaded as a separate file.

For peer review only
